# Efficient Purification of Cowpea Chlorotic Mottle Virus by a Novel Peptide Aptamer

**DOI:** 10.3390/v15030697

**Published:** 2023-03-07

**Authors:** Georg Tscheuschner, Marco Ponader, Christopher Raab, Prisca S. Weider, Reni Hartfiel, Jan Ole Kaufmann, Jule L. Völzke, Gaby Bosc-Bierne, Carsten Prinz, Timm Schwaar, Paul Andrle, Henriette Bäßler, Khoa Nguyen, Yanchen Zhu, Antonia S. J. S. Mey, Amr Mostafa, Ilko Bald, Michael G. Weller

**Affiliations:** 1 Federal Institute for Materials Research and Testing (BAM), 12489 Berlin, Germany; 2 Charité—Universitätsmedizin Berlin, Corporate Member of Freie Universität Berlin, Humboldt-Universität zu Berlin, and Berlin Institute of Health, 10117 Berlin, Germany; 3 Department of Diagnostic and Interventional Radiology, Technical University of Munich, 81675 Munich, Germany; 4 SAFIA Technologies GmbH, 12489 Berlin, Germany; 5 EaStCHEM School of Chemistry, University of Edinburgh, Edinburgh EH9 3FJ, UK; 6 Institute of Chemistry—Physical Chemistry, University of Potsdam, 14476 Potsdam, Germany

**Keywords:** cowpea chlorotic mottle virus, purification, affinity extraction, affinity chromatography, CCMV-binding peptide, virus-like particles, plant virus, nanotechnology, nanoparticles, virus production, safety issues, ultracentrifugation-free protocol, molecular dynamics

## Abstract

The cowpea chlorotic mottle virus (CCMV) is a plant virus explored as a nanotechnological platform. The robust self-assembly mechanism of its capsid protein allows for drug encapsulation and targeted delivery. Additionally, the capsid nanoparticle can be used as a programmable platform to display different molecular moieties. In view of future applications, efficient production and purification of plant viruses are key steps. In established protocols, the need for ultracentrifugation is a significant limitation due to cost, difficult scalability, and safety issues. In addition, the purity of the final virus isolate often remains unclear. Here, an advanced protocol for the purification of the CCMV from infected plant tissue was developed, focusing on efficiency, economy, and final purity. The protocol involves precipitation with PEG 8000, followed by affinity extraction using a novel peptide aptamer. The efficiency of the protocol was validated using size exclusion chromatography, MALDI-TOF mass spectrometry, reversed-phase HPLC, and sandwich immunoassay. Furthermore, it was demonstrated that the final eluate of the affinity column is of exceptional purity (98.4%) determined by HPLC and detection at 220 nm. The scale-up of our proposed method seems to be straightforward, which opens the way to the large-scale production of such nanomaterials. This highly improved protocol may facilitate the use and implementation of plant viruses as nanotechnological platforms for in vitro and in vivo applications.

## 1. Introduction

Plant viruses and their respective virus-like particles (VLP) offer an exciting nanotechnological platform for a variety of applications. Their propensity to self-assemble into monodisperse and highly symmetric nanometer-range particles makes them obvious candidates for the entrapment and delivery of biomedically active cargos in vivo [1,2,3,4,5,6,7,8,9,10]. In addition, the rigid protein capsids may be genetically or chemically modified to introduce (bio)chemical moieties to be displayed on the viruses’ surface [11,12,13,14,15,16,17]. The resulting conjugates exhibit strong polyvalency while preserving the bioavailability of these nanomolecular building blocks and are used as carriers for the presentation of antigens or other biochemically relevant units [2,18].

**Figure 1:**
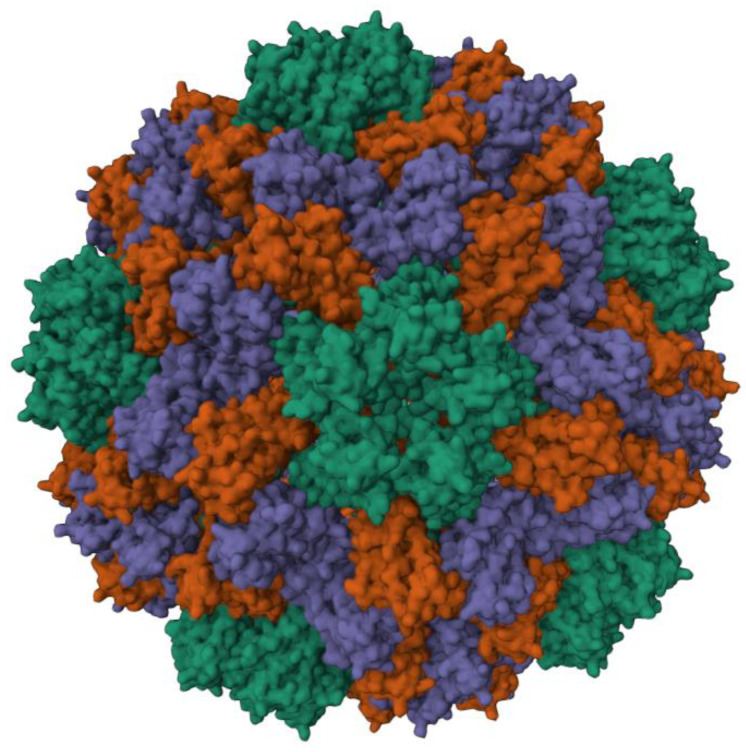
3D model of the capsid of CCMV (PDB ID: 1ZA7) generated with X-ray crystallography data from the RCSB protein data bank [31], created with Mol* Viewer [22]. The 180 subunits of the virus consist of chemically identical proteins with three types of different symmetries, highlighted in green, red, and purple, respectively. Please note that the model is created using high-resolution X-ray crystallography data of a K42R CCMV mutant.

The cowpea chlorotic mottle virus (CCMV) is a plant virus from the family *Bromoviridae*, and its particles have an icosahedral symmetry with a diameter of 28 nm [19,20]. The capsid consists of 180 identical copies of the coat protein [21]. A 3D model is shown in Figure 1 [21,22]. The CCMV can be produced with high yields of 0.1–1 mg per gram of infected leaf tissue [23]. Hence, CCMV is an attractive building block in many scientific fields. For example, Lam et al. used CCMV as a carrier for gene therapy to deliver siRNAs [24]. Apart from heterologous RNAs, a wide range of other non-viral cargo has also been packaged by self-assembled viral capsid proteins of CCMV; for example, anionic polymers [25], mineralized salts [26], gold nanoparticles [27], and fluorescent proteins [28]. In addition, CCMV VLPs were recently studied as potential candidates for in situ cancer vaccines [29] and scaffolds to display SARS-CoV-2 antigens [30].

The success of future applications of this nanotechnological platform, however, strongly depends on the availability and purity of the starting material. So far, native CCMV is propagated in the host plant, *Vigna unguiculata* (cowpea), and isolated by repeated precipitation with polyethylene glycol (MW 8000) and NaCl [32,33]. After resuspension of the second pellet containing the enriched but still impure CCMV, a final polishing step is necessary—usually density gradient ultracentrifugation [23,34] and sometimes ultrafiltration [35]. It should be noted that ultracentrifuges are quite expensive equipment, poorly suited for upscaling, and related to severe safety issues [36]. The availability of an appropriate ultracentrifuge often limits access to purified virus preparations. In addition, the purity of the isolated CCMV and the characterization of the purification steps have not been the subject of in-depth studies. Applying plant viruses in vivo demands the highest standards to avoid any unwanted contamination. Virus-like particles (VLP) of CCMV are commonly produced recombinantly [37,38]. A Coomassie-stained SDS-PAGE gel is usually considered to be sufficient for characterizing the eluate of the coat proteins after Ni-NTA purification (IMAC).

**Figure 2:**
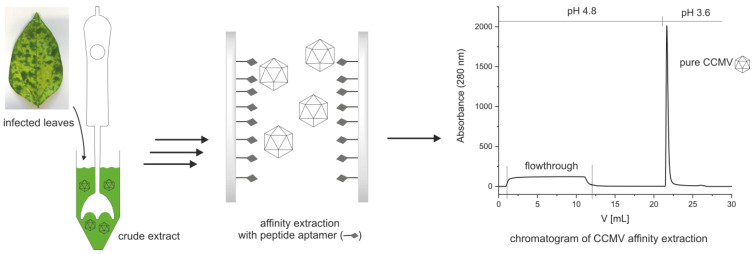
Simplified workflow for the purification of CCMV using a novel peptide aptamer.

To improve the availability and purity of CCMV, we developed a new two-step method for the purification of CCMV from infected leaves (see Figure 2). After propagation of CCMV in its natural host, *Vigna unguiculata*, symptomatic leaves are harvested and homogenized to a crude extract. Then, the CCMV is precipitated once using PEG 8000. Next, the resuspended CCMV is purified by affinity extraction and subsequent elution on a novel CCMV-binding peptide aptamer immobilized on a monolithic glass column.

This powerful and fast purification step eliminates the need for multiple PEG precipitations or any density gradient ultracentrifugation. Although RNA and DNA aptamers have been used in the past in order to detect other viruses [39,40,41], the use of peptide aptamers for the purification of plant viruses is a new concept. In our protocol, we qualified the product impurities at each purification step using, among other methods, silver-stained SDS-PAGE, MALDI-TOF MS, and size exclusion chromatography combined with UV detection. Furthermore, the purity of the virus isolates was quantitatively determined by reversed-phase HPLC/UV, and the total yield of CCMV in each step was calculated using enzyme-linked immunosorbent assay (ELISA) based on a new monoclonal antibody.

## 2. Materials and Methods

### 2.1. Biochemicals and Other Reagents

Buffers: PBS (10× powder, from AppliChem (Darmstadt, Germany), A0965,9010); Acetate binding buffer: sodium acetate (30 mM), acetic acid (20 mM), Na_2_-EDTA (1 mM), pH 4.8; Acetate elution buffer: acetic acid (6 mM), Na_2_-EDTA (1 mM), pH 3.6; Acetate neutralization buffer: sodium acetate (584 mM), acetic acid (292 mM); SEC running buffer: Sodium acetate (30 mM), acetic acid (20 mM), sodium chloride (150 mM), Na_2_-EDTA (1 mM), pH 4.8; phosphate binding buffer: Na_2_HPO_4_ · 2 H_2_O (8.7 mM), NaH_2_PO_4_ · 2 H_2_O (3.3 mM), pH 7.4; phosphate elution buffer: NaH_2_PO_4_ · 2 H_2_O (6.0 mM), H_3_PO_4_ (6.0 mM), pH 2.3; 2× loading buffer for SDS-PAGE (reducing): Glycerol (24%), tris base (900 mM, pH 8.45) (72%), sodium dodecyl sulfate SDS (4%), TCEP (100 mM), bromophenol blue (0.02%); 1× SDS running buffer: tris base (100 mM), tricine (100 mM), SDS (1%); and Solvents: Ethanol (absolute for HPLC, Th. Geyer (Renningen, Germany), 2222).

Other reagents: Acetic acid (Th. Geyer (Renningen, Germany), 2289-1L); EDTA disodium salt 2-hydrate (AppliChem (Darmstadt, Germany), 131669.1209); formaldehyde (Sigma Aldrich (St. Louis, MO, USA), 252549-25ML); (3-glycidyloxypropyl)trimethoxysilane (Sigma-Aldrich (St. Louis, MO, USA), 440167, CAS 2530-83-8); hydrochloric acid (Th. Geyer (Renningen, Germany), 857,1011); Mucasol ^®^ (Th. Geyer (Renningen, Germany), 230091); polyethylene glycol MW 8000 (Sigma-Aldrich (St. Louis, MO, USA), P2139); potassium dihydrogen phosphate (Th. Geyer (Renningen, Germany), 1648.0250); silver nitrate (Roth (Karlsruhe, Germany), 9370.4); sodium acetate (Th. Geyer (Renningen, Germany), 8694-1KG); sodium carbonate (AppliChem (Darmstadt, Germany), AP141648.1211); sodium chloride Thermo Fisher Scientific (Waltham, MA, USA), 446212500; sodium hydroxide (Sigma-Aldrich (St. Louis, MO, USA), 30620-1KG-R; sodium thiosulfate Roth (Karlsruhe, Germany), HN25.1); and Triton X-100 Sigma-Aldrich (St. Louis, MO, USA), T8787-50ML), 37470.01. Water was purified by an Ultra-Pure Water System from Millipore Co., (Burlington, MA, USA), with a resistivity of 18.2 MΩ·cm.

Antibodies: Anti-rabbit antibody (Origene (Rockville, MD, USA), R1364P); anti-mouse antibody (Jackson ImmunoResearch (Ely, UK), 111-005-008); HRP-anti-mouse antibody (115-035-003); and HRP-anti-rabbit antibody (Jackson ImmunoResearch, 111-035-003).

Virus: A purified sample of CCMV was kindly supplied by Dr. Guillaume Tresset (Université Paris-Saclay, Gif-sur-Yvette, France). The virus preparation was delivered in 50 mM acetate buffer of pH 4.8, with a concentration of 3 mg/mL.

Preparation of the monolithic raw column: The sintered core monolith (Por. 5, ultrafine, pore size 1–1.6 µm, borosilicate glass 3.3) was obtained from ROBU Glasfilter-Geräte GmbH, Hattert, Germany. The monolithic cylinder is prepared by custom order with the designation VitraPOR 5, diameter 8 mm, length 35 mm, and front surfaces finely sawn. The raw cylinder is glued into a titanium tube with the outer dimensions of 35 mm × 12 mm and a wall thickness of 1 mm using silicon F liquid, obtainable from Weicon (Münster, Germany), 13200310. A detailed description of the construction and manufacturing of the column and the column holder is shown in previous work [42]. In this work, the monolith length was increased from 15 mm to 35 mm.

### 2.2. Generation of a Peptide Binder for CCMV

#### 2.2.1. Experimental Peptide Binder Generation

For the screening, a one-bead-one-compound (OBOC) library with a ladder sequence was synthesized as previously reported [43]. The peptide library was synthesized by attaching the 4-hydroxybenzoic acid linker to TentaGel-resin. A short spacer comprising of Gly-Gly-Thr-Glu-Arg-Ser-Gly-Gly was added. Octamer peptides with a ladder sequence were coupled onto the spacer via the split-mix method [44]. The library contained glycine, serine, proline, tyrosine, glutamine, isoleucine, phenylalanine, tryptophane, histidine, glutamic acid, and arginine to cover hydrophilic, hydrophobic, basic, acidic, and aromatic amino acids. Similar amino acid compositions have previously proved suitable for the generation of peptide libraries [45]. The beads were immobilized on an electrically conductive, double-sided adhesive tape attached to a glass microscope slide. Details of the chip creation can be found in previous works [43].

Fluorescently labeled CCMV was made by mixing 95 µL of a solution of CCMV in PBS (1 g/L) and 5 µL of NHS-Dy-654 (5 g/L) under dark conditions for 1 h at 22 °C. The solution was purified by using a Vivaspin500 (Sartorius, Göttingen, Germany, VS0141) filter. The solution was mixed with 400 µL acetate buffer (0.2 M, pH 4.8) and centrifuged for 5 min at 12,000× *g*. Then, 450 µL of the acetate buffer was added, and the solution was again centrifuged for 5 min at 12,000× *g*. The step was repeated two times. The coupling success was examined via UV spectroscopy and MALDI-TOF MS.

The chip was pre-incubated in 15 mL acetate buffer for 1 h, and the buffer was removed. Afterward, a virus-free plant extract (300 mg of mashed leaves in 2 mL 0.2 M sodium acetate buffer pH 4.8; centrifuged at 25,000× *g* at 4 °C and filtered by 0.2 µm) was added and incubated for 2 h. The solution was removed, and the chip was washed three times with acetate buffer, dried for 30 min under air, and scanned with a microarray scanner Axon GenePix 4300A (Axon Instruments, Molecular Devices, San Jose, CA, USA) at 635 nm. After this pre-scan, the chip was again incubated in acetate buffer for 1 h. Following that, 5.17 µL of the CCMV-Dy654 conjugate was mixed with 15 mL of the plant extract, and the chip was incubated under dark conditions overnight. The solution was removed, and the chip was washed three times with acetate buffer, dried for 30 min under air, and scanned again with the microarray scanner at 635 nm.

The plant matrix was removed from the chip by incubation of 6 M guanidine HCl solution for 20 min. The chip was washed with 0.1% TFA in double deionized water, acetate buffer, 0.3% ammonium dodecyl sulfate in double deionized water, double deionized water, double deionized water:EtOH:TFA (94.9%:5%:0.1%), and double deionized water. The peptides were cleaved in a humid ammonia gas atmosphere overnight, and a 2,5-DHAP matrix was applied via an airbrush system, as previously reported [43]. The coordinates of the positive particles on the chip from the fluorescence scan were used for aiming with the laser in the MALDI-TOF MS (Bruker (Billerica, MA, USA) Autoflex maX smartbeam2) measurement.

The identified peptide was resynthesized by using the Titan 357 peptide synthesizer (AAPPTec, Louisville, KY, USA). For this, 100 mg of Fmoc-Rink Amide aminomethyl resin (Iris Biotech GmbH, Marktredwitz, Germany, 0.59 mmol/g) was placed into a reaction vessel. The Fmoc protecting group was cleaved by applying 2.5 mL of cleaving solution (20% piperidine in DMF) and shaking for 5 min. The solution was removed, and another 2.5 mL of cleaving solution was added for 10 min shaking. The resin was washed five times with 2 mL DMF for 1 min each wash. After removal of the last washing solution, 1 mL of amino acid with oxyma (both 0.4 M in DMF) and 1 mL of DIC (0.4 m in DMF) were added to the resin and shaken for 1 h. The coupling solution was removed, and the resin was washed once with 2 mL DMF for 1 min. Again, the amino acid/oxyma solution was added (1 mL) for the second coupling round. This time, 1 mL HCTU (0.4 M in DMF) and 1 mL of DIPEA (1 M in DMF) were added to the resin-amino-acid-slurry and shaken for 1 h. The solution was removed, and the resin was washed three times with 2 mL DMF for 1 min each wash. Again, Fmoc was removed, as described before. Finally, the resin was washed two times with EtOH (2 mL), and the particles were placed in a syringe reactor. Then, 4 mL of agent K (83% TFA, 5% double deionized water, 5% 1,2-ethanedithiol, 5% thioanisol, and 2% triisopropylsilane) were added to the resin for 3 h. The solution was poured into cold diethyl ether, and the precipitate was collected by centrifugation at 3280× *g*. The precipitate was washed once with diethyl ether and dissolved with DMSO. After lyophilization, a product of 75 mg was obtained. The crude peptide was used without further purification.

#### 2.2.2. Molecular Dynamics Simulations of the Peptide with GROMACS

GROMACS v.2022, a molecular dynamics (MD) simulation tool, was used to model the equilibrium properties of the aptamer peptide in solution [46]. The following outlines the modeling pipeline: As a starting point, an initial model of the peptide and linker was generated with Alphafold2 (v2.3.0, AF2) [47]. The modeled peptide of rank 1 was then processed for MD simulations to obtain an equilibrium structure in solution. The peptide’s coordinates were solvated in a dodecahedron box of TIP3P water molecules using periodic boundary conditions. A minimum distance between the solute and the boundary was set to 1 nm, and the AMBER99SB forcefield [48] was applied to the peptide. GROMACS took the protonation state of the peptide free in a solvent at pH 7, which should be different from that at pH 4.8. No ion was added to the system as the dilute buffer ions were unlikely to interact with the peptide. The system was energy-minimized with 500 steps of the steepest descent algorithm. Then, heavy peptide atoms were position-restrained with harmonic force constants of 1000 kJ mol^−1^ nm^−2^, while water molecules were allowed to equilibrate in an NVT ensemble for 200 ps followed by an NPT equilibration for a further 200 ps using the Leap-Frog algorithm [49]. The mean temperature of 300 K was achieved using the velocity-rescaling thermostat [50] with a time constant of 0.1 ps. The pressure was maintained at 1 bar using the Parrinello–Rahman barostat [51] with a time constant of 2 ps. Short-range nonbonded interactions were truncated using a Verlet cut-off scheme at 10 Å. The smooth particle-mesh Ewald [52] was used to calculate long-range electrostatics. The production simulation consisted of 5 parallel 1 μs simulations using a timestep of 2 fs, with frames saved every 40 ps. All input files for the simulations can be found at: https://github.com/meyresearch/CCMV_aptamer_msm (accessed on 28 February 2023).

#### 2.2.3. Markov Modeling to Obtain the Dominant Equilibrium Peptide Structure in Solution

In order to analyze the 5 μs of the trajectory data, a Markov State model (MSM) was built using pyEMMA v2.5.7 [53] following the pyEMMA tutorial [54] and similar approaches [55,56]. The trajectories were featured using backbone torsion angles and distances between α-carbon atoms. Time-lagged independent component analysis (TICA) [57] was performed to reduce the dimension of the feature space, which then produced a 64-dimensional subspace using a 95% kinetic variance cut-off. The TICA space was discretized using a k-means algorithm with 300 cluster centers. The maximum likelihood MSM was estimated from the discrete trajectories using a lag time of 1 ns (see Figure S1). The PCCA+ algorithm [58] was used to coarse-grain the MSM into two metastable states. The most likely state was identified as the dominant equilibrium peptide structure. The final visualization of the equilibrium structure rendered using VMD v.1.9.3 [59] was obtained by drawing random samples from the most populated PCCA state. A Jupyter notebook containing the MSM analysis is provided at: https://github.com/meyresearch/CCMV_aptamer_msm (accessed on 28 February 2023).

### 2.3. Preparation of the Affinity Column with CCMV-Binding Peptide

An assembled column system consisting of the monolithic column [42] built in a 3D-printed column holder was connected to a syringe pump (Standard Infusion Only Pump 11 Elite from Harvard Apparatus, Holliston, MA, USA), and several reagents and washing solutions were pumped through the column. If not indicated otherwise, a flow rate of 1 mL/min was used, and the reactions took place at room temperature.

To clean the glass surface, 1 mL/min 10 mL of Mucasol (1%), NaOH (10%), and HCl (10%) were pumped through the column subsequently. Afterward, the column was washed with 10 mL of PBS at 1 mL/min to obtain a neutral pH before the column was equilibrated with 10 mL of toluene. In the first reaction step, 10 mL of (3-glycidyloxypropyl)trimethoxysilane (10% in toluene) was pumped through the column within 10 min. The column was sealed on both sides and incubated for 1 h at room temperature. After the reaction took place, the column was manually flushed with an air-filled syringe to remove the remaining silane solution from the column. The monolithic column was then removed from its column holder and placed in a drying oven (UNB 200, Memmert, Schwabach, Germany) to incubate overnight at 130 °C. Then, the column was allowed to cool down for 20 min and put back in the column holder. After flushing the reassembled column with 10 mL of toluene at 1 mL/min to remove the left-over silane, the column was again flushed with air to remove any remaining toluene. The column surface was now presenting epoxide groups and was ready for coupling the column with the target peptide via its thiol group.

For the peptide coupling, 12 mg of the CCMV-binding peptide was dissolved in 7.2 mL of DMSO. While gently vortexing the peptide solution, 4.8 mL of a Tris buffer (100 mM, pH 9.0) was added slowly. The acquired 12 mL of ready-to-couple peptide solution was now pumped through the column within 1 h at room temperature. To block the remaining epoxy groups, the column was flushed with 10 mL of a cysteine solution (100 mM in Tris buffer (100 mM, pH 9.0)) within 1 h. Next, the column was flushed at a flowrate of 1 mL/min with 10 mL of a Tris buffer (100 mM, pH 9.0), 10 mL of H_2_O, and eventually washed with 10 mL of EtOH (20% in H_2_O) and stored in a fridge until further use.

### 2.4. Optimized Protocol for the Purification of CCMV

The cowpea chlorotic mottle virus (CCMV) isolate was kindly supplied by Dr. Guillaume Tresset (Université Paris-Saclay, France). CCMV was grown in leaves of *Vigna unguiculata* (cowpeas, black-eyed peas). For the germination of the seeds and growth of the plants, a hydroponic system with in-built and automated LED illumination from iDOO (Model: ID-IG301, Eastvale, CA, USA) was used. The first true leaves of the plants (not the cotyledons) were inoculated with a 50 µL suspension of 0.1 mg/mL CCMV in 0.05 M sodium acetate buffer and 1 mM Na_2_EDTA, pH 4.8 and a spatula tip of silicon carbide powder (carborundum, 600 grit) by gentle but firm rubbing of the leaves’ surface. After 3 min, excess silicon carbide was rinsed off with lab water. After 7–14 days, symptomatic leaves were harvested, weighed, and stored at −20 °C in a plastic bag. Any further symptomatic regrown leaves were also harvested and stored at −20 °C in a plastic bag.

The infected plant material was homogenized with a standard household blender (Bosch VitaBoost MMBH6P6BDE, Gerlingen, Germany). For every gram of leaves, 6 mL of extraction buffer (0.2 M sodium acetate buffer with 10 mM Na_2_-EDTA and 0.1% (*w*/*v*) ascorbic acid, pH 4.8) was used. After 1 min of blending, 1 droplet of defoamer (neodisher Entschäumer S, article number: 430148) was added and mixed with the extract for 3 s. The extract was transferred into 50 mL falcon tubes and centrifuged at 15,000× *g* and 4 °C for 20 min, then filtered with a 0.2 µm syringe filter. A final concentration of 10% (*w*/*v*) PEG 8000 was added to the filtrate and thoroughly vortexed. Afterward, the solution was placed in an overhead shaker and allowed to incubate at 4 °C overnight. After incubation, the solution was centrifuged at 15,000× *g* and 4 °C for 25 min to obtain a yellow pellet. The supernatant was discarded. The pellet was completely resuspended in binding buffer (0.05 M sodium acetate buffer with 1 mM Na_2_-EDTA, pH 4.8) using a quarter of the initial volume by thorough vortexing. A few seconds of ultrasonication can help in the resuspension process. After resuspension, a pellet is again formed by centrifuging at 15,000× *g* and 4 °C for 15 min. This pellet does not contain CCMV. The supernatant is filtered with a 0.2 µm syringe filter and used for the affinity extraction performed on an FPLC system (ÄKTA pure 25 L, Cytiva Life Sciences, Marlborough, MA, USA) using the peptide aptamer column described in Section 2.2.

Before each run on the FPLC, the column was washed with 20 mL of 1% Triton X-100 solution, followed by washing with elution buffer (6 mM acetic acid, 1 mM Na_2_-EDTA, pH 3.6), and a subsequent equilibration with lab water and then binding buffer (0.05 M sodium acetate buffer with 1 mM Na_2_EDTA, pH 4.8). The fractions of the fraction collector were each filled with 10 µL of neutralization buffer (292 mM acetic acid, 584 mM sodium acetate, pH 4.5). Then, the sample loop (10 mL) was filled with the sample. During the injection, a flow of 2 mL/min was applied. Afterward, a washing step with 10 mL binding buffer and a flow rate of 10 mL/min was used. Finally, the CCMV was eluted with the elution buffer (6 mM acetic acid, 1 mM Na_2_EDTA, pH 3.6) at a flow rate of 6 mL/min and collected in 200 µL fractions in the fraction collector. The neutralized fractions—now at 0.05 M sodium acetate buffer with 1 mM Na_2_EDTA, pH 4.8—were pooled so that ~95% of the peak area was saved without unnecessary dilution of the sample and stored at 4 °C for later analysis. An aliquot was used to determine the concentration of the CCMV by UV absorbance, using the extinction coefficient of CCMV at 260 nm: ε_260_ = 5.87 mg^−1^ mL cm^−1^ [60]. The column was re-equilibrated with binding buffer and stored at 20% ethanol at 4 °C.

### 2.5. Characterization of Purification Steps with Silver-Stained SDS-PAGE

From samples 1, 3, 4 and 5, 10 µL aliquots were taken and each supplemented with 10 µL of 2 × SDS loading buffer, mixed, and heated to 95 °C in a ThermoMixer C (Eppendorf, 5382000015) for 15 min. The samples were mixed again and, subsequently, 10 µL of each sample was loaded onto the gel (Novex™ WedgeWell™ 8–16%, Tris-Glycine, 1.0 mm, Mini Protein Gel, Invitrogen, XP08160BOX, Waltham, MA, USA), which was run with 900 mL of SDS running buffer in a fridge at 4 °C with a XCell SureLock Mini-Cell Electrophoresis System from Invitrogen (EI0001). Run times were 20 min at 80 V + 25 min at 200 V. The following silver-staining protocol was applied to visualize protein bands: The gel was washed twice in lab water for 2 min each wash. Afterward, the gel was incubated in sodium thiosulfate Na_2_S_2_O_3_ (1.3 mM) for 1 min and then washed with water for 10 s. The gel was then transferred into a staining container containing freshly prepared AgNO_3_ (5.9 mM) and incubated for 25 min. The gel was washed twice with water for 10 s each wash, and then incubated for 30 s with sodium carbonate Na_2_CO_3_ (236 mM). In the next step, the gel was incubated in a solution of Na_2_CO_3_ (236 mM) and formaldehyde (2.5 mM) for 7 min. After the completed staining, the gel was washed twice with water and then incubated in Na_2_EDTA (50 mM) for 10 min. Last, the gel was washed twice with water, and a photograph of the final gel was taken.

### 2.6. Characterization of Purification Steps with Size Exclusion Chromatography (SEC)

Size exclusion chromatography was performed on an FPLC (ÄKTA pure 25 L, Cytiva Life Sciences, Marlborough, MA, USA); 280 nm was used as the detection wavelength. A flow rate of 2.6 mL/min was used during the washing steps and 0.5 mL/min during separations. The experiment was performed with samples 1, 3, 4, and 5, subsequently. To each of the four samples, 150 mM sodium chloride was added. The CCMV eluate (sample 5) was diluted in SEC running buffer to a concentration of 100 µg/mL. The obtained samples were filtered with a 0.22 µm syringe filter. The column (Hi Prep 26/60 Sephacryl S-300-HR, from Cytiva, Marlborough, MA, USA, 17119601) was connected to the FPLC system and then equilibrated with 1.5 column volumes (CV) water and SEC running buffer, subsequently. During the applied purification protocol, the column was first equilibrated with 0.1 CV of SEC running buffer before the 1 mL sample was injected through the 2 mL sample loop using 10 mL of SEC running buffer. Afterward, the column was eluted with 1.5 CV of SEC running buffer, where the separation took place.

### 2.7. Characterization of Purification Steps with MALDI-TOF MS

Samples 1, 3, 4, and 5 were desalted using Zeba Spin 7K MWCO (89878, Th. Geyer, Renningen, Germany) size-exclusion desalting columns (75 µL) according to the manufacturer’s protocol. Then, 1 µL of the eluted solution was pipetted on a target spot of the MALDI plate, and 1 µL of α-cyano-4-hydroxycinnamic acid (10 mg/mL in 50% H_2_O, 49.9% ACN, 0.1% TFA (*v*/*v*/*v*)) was added. The droplet was mixed by pipetting up and down several times and then allowed to dry. MALDI-TOF MS was performed on a Bruker (Billerica, MA, USA) Autoflex maX smartbeam2 in linear mode. The obtained spectra were averaged over 5000 laser shots.

### 2.8. Purity Determination by Reversed-Phase HPLC

The eluate of the affinity extraction (sample 5) was diluted with lab water to a concentration of 0.3 mg/mL and filtered with a 0.1 µm syringe filter into an HPLC vial. A high-performance liquid chromatography system with a diode array detector (HPLC-DAD; Agilent Technologies 1260 Infinity II series, Santa Clara, CA, USA) was used. The injection volume was 30 µL. A C_8_ column (AdvanceBio RP-mAb SB-C8, 2.1 × 150 mm, 3.5 µm, Agilent Technologies) was used as the stationary phase. Separation was performed with A = 0.2% TFA in H_2_O and B = 0.16% TFA in acetonitrile with the following gradient—0–3 min 99% A and 1% B; 3–23 min 30% A and 70% B; 23–40 min 1% A and 99% B—at a constant flow rate of 0.8 mL/min. Baseline subtraction, peak finding, and integration were performed using Chromeleon chromatographic data analysis software (version 7.2.10.23925).

### 2.9. Determination of Integrity and Monodispersity

Transmission electron microscopy (TEM) images were obtained in a Talos F200S Microscope (Thermo Fisher Scientific, Waltham, MA, USA) by using a 200 kV microscopy technique in which a beam of electrons is transmitted through a specimen to form an image. The specimens were prepared by negative staining the CCMV with a 3% solution (*w*/*v*) of phospho-tungstic acid in ultrapure water onto a 3 mm copper grid (lacey, 400 mesh, TED PELLA, Redding, CA, USA, 01824) using the side-blot method.

Dynamic light scattering experiments were performed with a Zetasizer Malvern Instrument with disposable cuvettes made from polystyrene (10 mm) purchased from Th. Geyer GmbH & Co., KG (Renningen, Germany). Measurements were performed in forward scatter mode and the advanced cumulant model. A 1 mg/mL CCMV solution in 0.05 M acetate buffer containing 1 mM EDTA, pH 4.8, was freshly prepared and measured.

Atomic force microscopy (AFM) measurements were performed on a HORIBA OmegaScope with LabRAM HR evolution instrument, using tapping mode (AC mode) with ATEC-NC (frequency 335 kHz, spring constant 45 N/m) tips. The AC mode automatically controls all the parameters with the exception of the scan rate which was set to 1 Hz. Then, a 1 µL 0.1 mg/mL CCMV solution was added to 9 µL 1× Tris-acetate-EDTA buffer containing 15 mM MgCl_2_ and incubated on a plasma-treated silicon chip for 10 min. The chip was then washed two times with a mixture of ethanol and water (1:1) and blow dried with compressed air. The chip was taped on a magnetic disc and inserted in the AFM instrument for imaging.

### 2.10. Quantification of CCMV by ELISA

For the immunoassay, antiserum (BAM-CCMV-rab-pAb01) from rabbits after immunization with CCMV was purified via protein G affinity chromatography. The experiment was performed on an ÄKTA pure 25 L from GE Healthcare Life Sciences, and 280 nm was used as the detection wavelength. A flow rate of 4 mL/min was used if not mentioned otherwise. The rabbit serum was diluted 1:10 in a phosphate binding buffer (pH 7.4). The obtained serum solution (500 µL) was filtered using a 0.22 µm syringe filter and then filled into the 2 mL sample loop of the FPLC system. The column (HiTrap 1 mL Protein G HP, Cytiva, 29048581) was connected to the system and then washed with water, phosphate elution buffer, and phosphate binding buffer subsequently. During the applied method, the column was first equilibrated with 10 mL of binding buffer before the sample was injected with 6 mL of phosphate binding buffer at a flow rate of 0.5 mL/min through the sample loop. After a washing step with 15 mL of phosphate binding buffer to remove loosely bound components, IgG was eluted with 5 mL of phosphate elution buffer (pH 2.3). Finally, the column was re-equilibrated with 10 mL of phosphate binding buffer to a pH of 7.4. An IgG solution with 0.5 mg/mL in 600 µL was obtained and determined with UV absorption at 280 nm and rabbit IgG as a reference in a photometer (NanoPhotometer NP80, Implen, Munich, Germany).

For the determination of the CCMV with an enzyme-linked immunosorbent assay (ELISA), the protein G purified polyclonal antibody was used, as well as an anti-CCMV monoclonal antibody (BAM-CCMV-29-81) and secondary antibodies (polyclonal anti-mouse antibody and polyclonal peroxidase-conjugated anti-rabbit antibody). All steps were performed at room temperature, washing was done in triplicate using 300 µL of washing buffer (KH_2_PO_4_ 1.25 mM; Na_2_HPO_4_∙2H_2_O 6.67 mM; Tween 20 0.3 mM), and incubation was performed on a plate shaker. The high-binding microplate was coated in 100 µL of 1× PBS, pH 7.4, containing 2.6 µg/mL of anti-mouse antibody and incubated overnight. The next morning, the plate was washed, and 100 µL of the monoclonal anti-CCMV antibody at a concentration of 0.72 µg/mL in 1× PBS, pH 7.4, was added to the wells of the microplate. After one hour, the plate was washed and then incubated with 250 µL of the blocking solution (5% skimmed milk powder in 1× PBS, pH 7.4) for another hour. Following the next washing, the wells were treated with 100 µL of the sample dilutions (usually ranging from 1:100 to 1:50,000 depending on the supposed concentration) and the calibration solutions in PBS-T for an hour. Then, 100 µL of polyclonal anti-CCMV antibody (0.34 µg/mL) in PBS-T was added after washing, and the plate was again placed on the shaker for 1 h. The plate was washed, and 100 µL of the peroxidase-conjugated anti-rabbit antibody in PBS-T was added at a concentration of 2.67 ng/mL and incubated for 30 min. Then a final wash step was performed. For detection, 100 µL TMB substrate was added to the wells and incubated for 15 min in darkness. In the last step, 100 µL of stop solution (250 mM H_2_SO_4_) was added, and the absorbance at 450 nm was measured using the plate reader.

## 3. Results

### 3.1. Generation of a Peptide Aptamer for CCMV

**Figure 3:**
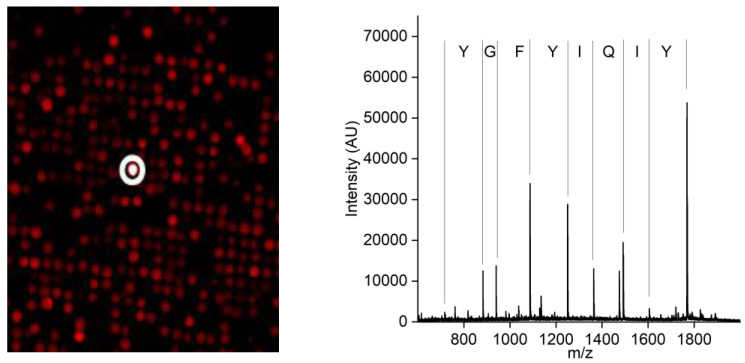
**Left**: A small image detail of the combinatoric peptide library immobilized on a glass slide after incubation with fluorescently labeled CCMV in a diluted extract of *Vigna unguiculata*. In total, approximately 25,000 different peptides were screened on one chip. The positive bead is highlighted with a white circle. **Right**: MALDI-TOF mass spectrum of the peptide after cleavage from the bead. The ladder sequence introduced during peptide synthesis allows for the simple readout of the peptide sequence without the need for fragmentation.

A synthetic peptide library containing linear peptides was used to screen for a CCMV-binding peptide. A positive hit in the screening resulted in the peptide with the amino acid sequence Tyr-Ile-Gln-Ile-Tyr-Phe-Gly-Tyr, which showed the most promising properties. The positive peptide bead is highlighted with a white circle in Figure 3 (left). The subsequent MALDI-TOF MS of the peptide candidate (Figure 3, right) revealed its sequence due to the introduced ladder sequence during peptide synthesis.

**Figure 4:**
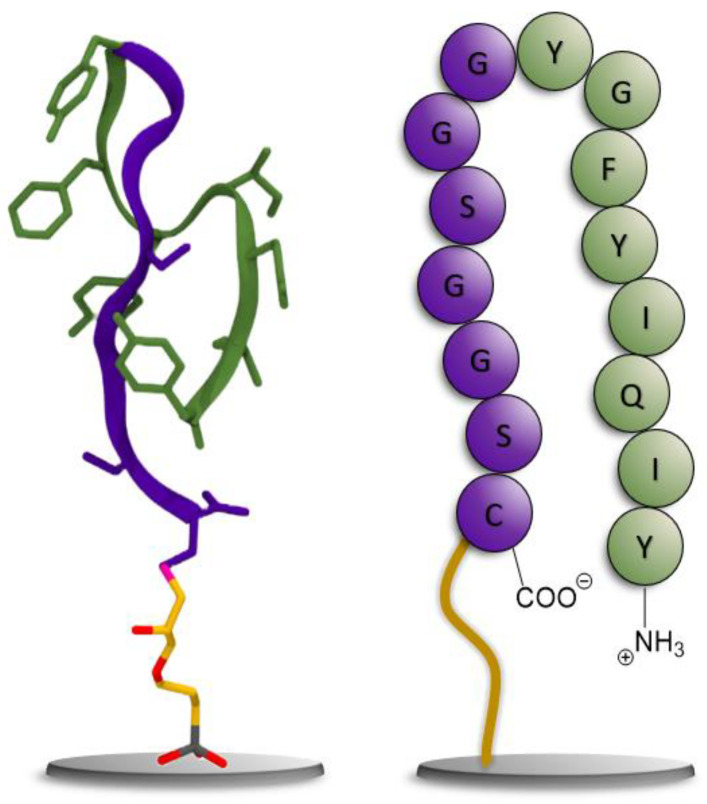
**Left**: Representative structure of the equilibrium-folded structure of the peptide; the glycine–serine linker is shown in purple, and the peptide aptamer in green, with the silane linker shown in licorice representation. **Right**: Cartoon representation of the peptide for comparison using the same color scheme.

For the resynthesis of the peptide, its C-terminus was extended with a glycine–serine linker for better solubility and steric reasons. In addition, a cysteine was added as the C-terminal amino acid to facilitate the immobilization of the peptide on a solid phase. To illustrate the peptide’s dominant folding behavior in water, a Markov State model (MSM) was used to extract the most stable folding structures from a molecular dynamics (MD) trajectory of 5 μs of the target peptide. The conformation shown on the left in Figure 4 shows a representative structure from an equilibrium ensemble observed at 96.6% from the MSM analysis of the MD simulations. The schematic on the right illustrates the corresponding amino acid sequence. For more details on the populations of peptide conformations from the MSM, see Figure S2.

### 3.2. Preparation of an Affinity Column with the CCMV-Binding Peptide Aptamer

**Figure 5:**
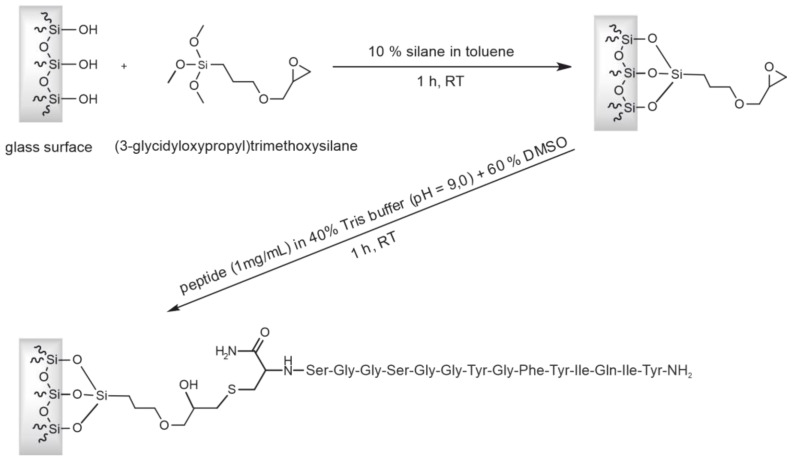
Scheme for preparing the affinity column for CCMV purification: Surface functionalization of a sintered glass monolith with epoxy silane and coupling of the thiol-containing CCMV-binding peptide aptamer via ring-opening of the epoxide. Please note: In this figure, the *N*-terminus of the peptide NH_2_-Tyr-Ile-Gln-Ile-Tyr-Phe-Gly-Tyr-Gly-Gly-Ser-Gly-Gly-Ser-Cys-NH_2_ is located on the right-hand side, and the *C*-terminus is amidated. Due to the specific synthesis and subsequent screening procedure, the peptide must be oriented so that the C-terminus is directed toward the solid phase.

For the preparation of the affinity column, sintered glass monoliths were used as the starting material for the immobilization of the CCMV-binding peptide. This material and the evaluation of its performance regarding affinity separations were previously published [42]. The detailed protocol for the treatment of the monolithic raw column, its assembly, and the cleaning of the glass surface was performed as described there. Afterward, the glass surface was functionalized with an epoxysilane, as shown in Figure 5. Then, 10 mL of 1 mg/mL of the CCMV-binding peptide in 40% Tris buffer (0.1 M, pH 9.0) and 60% DMSO were pumped through the column. The reaction of the thiolate from the C-terminal cysteine of the peptide and the epoxide can be considered a form of click-chemistry [61]. Therefore, the product should form quickly, with a high yield and essentially no byproducts. Afterward, the column was washed with DMSO, then water, before a cysteine-blocking solution was pumped through the column to block any unreacted epoxide groups.

**Figure 6:**
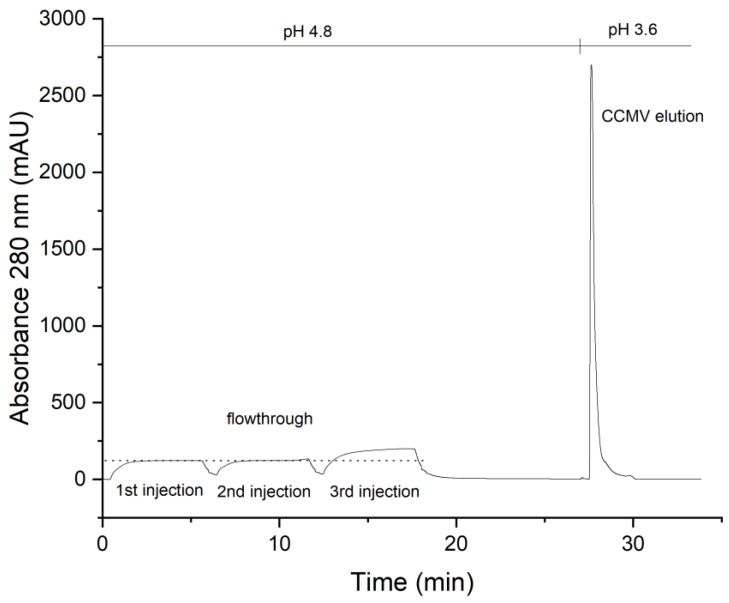
Column capacity for the binding of CCMV. Three 10 mL injections of a resuspended PEG-precipitate solution containing 0.1 mg/mL CCMV were made in succession. The flowthrough of the 1st and 2nd injections is free of any CCMV as determined with ELISA. It contains impurities from the plant matrix and PEG 8000. After the third injection, the column capacity is exceeded, and flowthrough peak is increased, as indicated by the dashed line. The column capacity was determined as approximately 2 mg of CCMV. After washing with binding buffer and elution at pH 3.6, pure CCMV was obtained as a narrow peak. The eluting CCMV is immediately neutralized to approximately 50 mM sodium acetate pH 4.8 with a small volume of concentrated neutralization buffer present in the fraction collector.

The capacity of the peptide column was determined by using an excess of CCMV with a known concentration in a plant extract. A maximum of 2 mg CCMV was bound by the column within 5 min (see Figure 6). The flowthrough consists of the plant matrix. The eluate of 1 mL was later measured by UV absorbance and yielded 2 mg/mL determined with the extinction coefficient of CCMV at 260 nm: ε_260_ = 5.87 mg^−1^ mL cm^−1^.

### 3.3. Optimized Protocol for the Purification of CCMV

**Figure 7:**
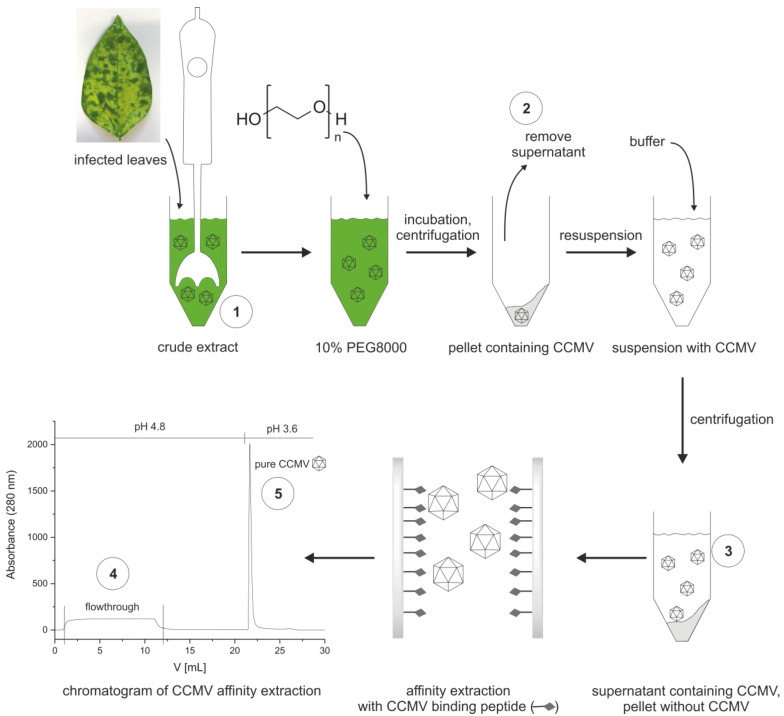
Optimized workflow for the purification of CCMV from leaves of *Vigna unguiculata*: Samples ① to ⑤ are taken from the different purification steps. ① Leaves are homogenized to a crude extract. The extract is centrifuged, and 10% PEG 8000 is added. After incubation overnight, the solution is centrifuged, and the supernatant ② is removed. The pellet containing CCMV is completely resuspended with buffer. Afterward, the suspension is centrifuged again to obtain the essentially CCMV-free pellet. The supernatant ③ containing the CCMV, among other proteins and PEG 8000, is filtered. Pure CCMV ⑤ is isolated from the supernatant by affinity extraction with the CCMV binding peptide immobilized on a sintered glass monolith surface; the flowthrough of column ④ is discarded.

Figure 7 shows the schematic workflow for the stepwise purification of the CCMV. First, a crude extract is made by blending infected leaves with extraction buffer (0.2 M sodium acetate buffer with 10 mM Na_2_-EDTA and 0.1% (*w*/*v*) ascorbic acid, pH 4.8). In our experience, 6 mL of extraction buffer for every gram of leaves is sufficient. The extract is centrifuged and filtered before PEG 8000 is added to a final concentration of 10% (*w*/*v*). The solution is allowed to incubate at 4 °C overnight before it is centrifuged again. The pellet formed contains the CCMV. The supernatant is discarded. The pellet is completely resuspended with binding buffer (0.05 M sodium acetate buffer with 1 mM Na_2_EDTA, pH 4.8). Afterward, the suspension is centrifuged, forming a pellet that does not contain the CCMV. Instead, the supernatant containing the CCMV, other plant matrix components, and PEG 8000 is filtered (0.2 µm) and used for the final purification step. In this final step, the CCMV is enriched on the affinity column shown in Figure 7. After washing the column with 10 column volumes of binding buffer, the CCMV is eluted from the column with 6 mM acetic acid, 1 mM Na_2_-EDTA, pH 3.6, and the elution peak is collected in 200 µL fractions. Each fraction in the fraction collector is prepared beforehand to contain 10 µL of neutralization buffer (292 mM acetic acid, 584 mM sodium acetate). Thus, the 200 µL fractions of CCMV eluates are immediately neutralized to 0.05 M sodium acetate, 1 mM EDTA, pH 4.8 upon reaching the fraction collector and can be stored at 4 °C.

From every purification step (① to ⑤), aliquots were taken and analyzed (when applicable) with SDS-PAGE, size exclusion chromatography (SEC), mass spectrometry, reversed-phase chromatography, transmission electron microscopy (TEM), atomic force microscopy (AFM), dynamic light scattering (DLS), and an immunoassay to determine the purity, integrity, and yield of the viral nanoparticles at these steps.

### 3.4. Characterization of Purification Steps with Silver-Stained SDS-PAGE

**Figure 8:**
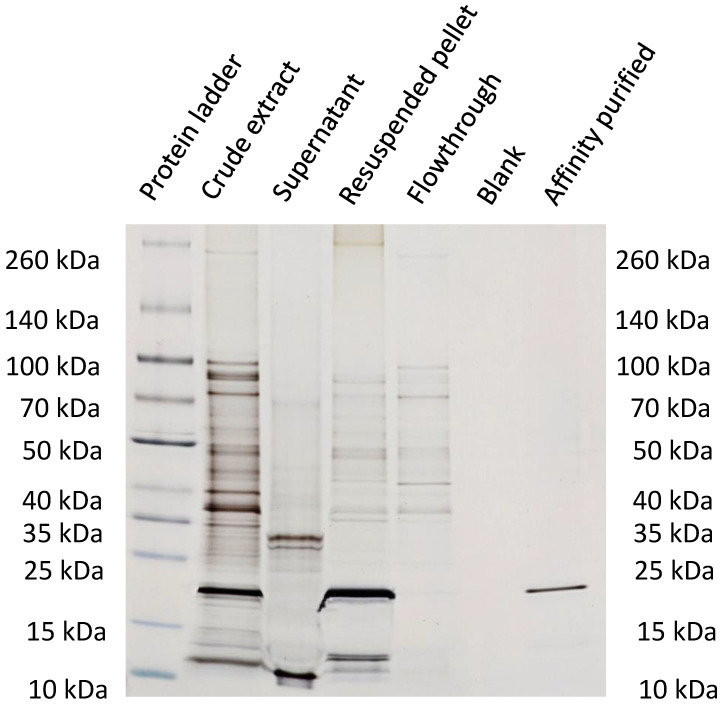
Silver-stained SDS-PAGE analysis of each purification step and the final eluate. Lane 1: Protein ladder; Lane 2: Crude extract; Lane 3: Supernatant after precipitation with PEG 8000; Lane 4: Supernatant of resuspended pellet; Lane 5: Flowthrough of affinity column; Lane 6: Binding buffer as blank; Lane 7: CCMV eluate obtained by affinity purification (final product, diluted to approx. 20 µg/mL).

The SDS-PAGE for samples ① to ⑤ (①—crude extract; ②—supernatant; ③—resuspended pellet; ④—flowthrough; ⑤—affinity purified virus) is shown in Figure 8. The CCMV is already detectable in sample 1, the crude extract, at around 20 kDa. The following pelleting step seems to be very efficient as no CCMV can be detected in the supernatant (sample ②), and CCMV could be enriched by PEG precipitation, as shown in the lane for sample ③. However, other protein impurities are still clearly visible. The PEG impurities cannot be detected by this method. The next lane demonstrates the need for the final affinity purification step as the flowthrough (sample ④) contains many of the impurities already detectable in sample ③. Sample ⑤ is the enriched CCMV eluate (1 mg/mL) diluted to 0.02 mg/mL, which does show any residual impurities.

### 3.5. Characterization of Purification Steps with Size Exclusion Chromatography

**Figure 9:**
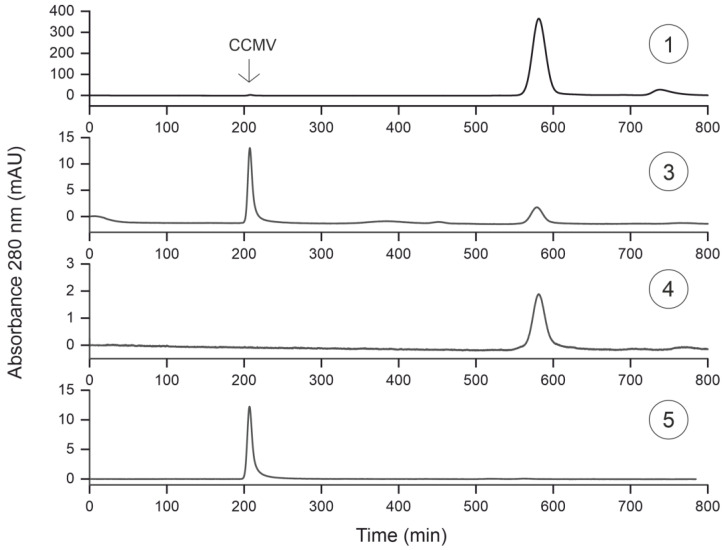
Size exclusion chromatograms (SEC) of samples taken at different steps of the purification protocol are shown in Figure 7: ① crude extract; ③ filtered supernatant after pelleting with PEG 8000; ④ flowthrough of the affinity extraction; ⑤ eluate of the affinity extraction. SEC was performed with a Hi Prep 26/60 Sephacryl S-300-HR, Cytiva (17119601); the running buffer was 0.05 M sodium acetate buffer, 0.15 M NaCl and 1 mM Na_2_EDTA, pH 4.8, with a flow rate of 0.5 mL/min.

Next, samples ①, ③, ④, and ⑤ were analyzed with size exclusion chromatography (SEC) using UV detection at 280 nm (Figure 9). Like the silver-stained SDS-PAGE gel, a small amount of CCMV is detectable in the crude extract (sample ①) at around 200 min. The smaller protein impurities elute later, around 590 min, and constitute the majority of the protein content in the extract. The void volume is only reached later for molecules smaller than the exclusion size of the column. Sample ③ contains the enriched CCMV after the pelleting step with PEG 8000. The chromatogram shows the virions as the dominant peak at 200 min. The purity of the CCMV based on the absorbance at 280 nm at this purification step can be calculated from the relative integrals of the eluting peaks. With this premise, the purity of the CCMV would be 71.1%. After the final affinity extraction (sample ⑤), the size exclusion chromatogram shows only the CCMV eluting at 200 min, and no other impurities could be detected. In contrast, the flowthrough of the affinity column (sample ④) contains impurities eluting at 590 min. The results from the SEC also indicate that the virions are still intact after the purification steps.

### 3.6. Characterization of Purification Steps with MALDI-TOF MS

**Figure 10:**
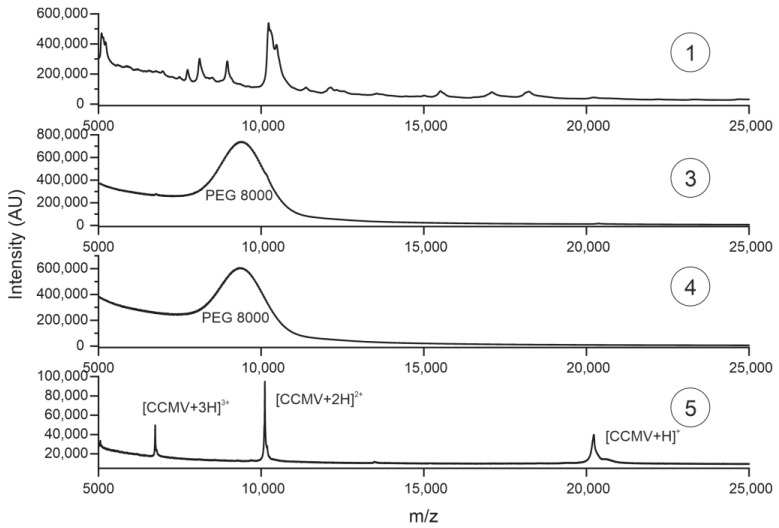
MALDI-TOF mass spectra of samples taken at different steps of the purification protocol are shown in Figure 7: ① Crude extract; ③ filtered supernatant after pelleting with PEG 8000; ④ flowthrough of the affinity extraction; ⑤ eluate of the affinity extraction.

It is important to note that the calculated purity is dependent on the detection method. Non-UV-absorbing molecules cannot be identified at 280 nm. For this reason, we analyzed samples ①, ③, ④, and ⑤ with matrix-assisted laser desorption/ionization time-of-flight mass spectrometry (MALDI-TOF MS) (see Figure 10). While the mass spectrum for sample ① shows the complexity of the plant matrix, the mass spectrum for sample ③, taken after the pelleting step, is even more noteworthy. Whereas the silver-stained SDS-PAGE gel shows CCMV as the dominant band and the SEC suggests a CCMV purity of >71%, the mass spectrum does not even contain the characteristic peak at ~20,300 Da corresponding to the CCMV capsid protein subunit. Instead, only PEG is detectable over a broad mass range with a maximum of 8000 Da. After the affinity extraction, the PEG is completely removed from the solution, as can be seen in the mass spectrum of sample ⑤, the eluate of the affinity extraction. The molecular ion peak at 20,300 Da [CCMV + H]^+^ and the peaks of the corresponding multiple charged species [CCMV + 2H]^2+^ and [CCMV + 3H]^3+^ are the dominant signals in the spectrum. The small peak at around 13,500 Da corresponds to the triply charged dimer [2CCMV + 3H]^3+^ of the capsid protein. No other impurities of higher molecular mass can be detected in the mass spectrum of the final eluate.

In some other samples of CCMV, however, we found multiple smaller peaks at *m/z* ratios of around 18,000–20,000 Da and their multiply charged species. Indeed, we could prove by peptide mass fingerprinting and western blotting that these peaks are fragments of the CCMV capsid protein (Supplementary Materials Figure S3). Furthermore, we observed a dependency on the age of the leaves that may indicate the degree of capsid protein degradation (Supplementary Materials Figure S4). The CCMV isolated from leaves harvested that were older than 6 weeks had far more degradation products than leaves harvested after only 1–3 weeks. We assume that the breakdown of the capsid protein may be caused by proteolytic cleavage. We tried to prevent proteolytic breakdown by the addition of plant protease inhibitors to the crude extract, but no difference in CCMV capsid protein degradation in older leaves could be noticed. For this reason, we concluded that the degradation is apparently already taking place in the plant.

### 3.7. Purity Determination by Reversed-Phase Liquid Chromatography (HPLC)

**Figure 11:**
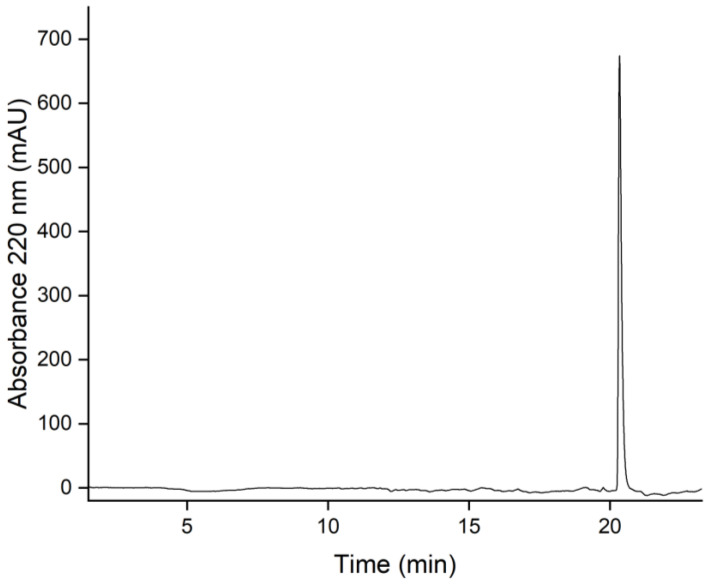
Reversed-phase high-performance liquid chromatography (RP-HPLC) of CCMV after purification by affinity chromatography. 9 µg CCMV was injected and subsequently separated using the following gradients: 0–3 min 99% A (H_2_O with 0.2% TFA) and 1% B (ACN with 0.16% TFA); 3–23 min 30% A and 70% B; 23–40 min 1% A and 99% B, at a constant flow rate of 0.8 mL/min.

The purity assessment of biomolecules is often performed using reversed-phase liquid chromatography with UV detection, e.g., at 220 nm [62]. At this wavelength, peptidic amide bonds have a strong absorbance. Figure 11 shows the chromatogram of sample 5 using a C8 column on an HPLC. Gradient separation with A: H_2_O with 0.2% TFA and B: Acetonitrile with 0.16% TFA was applied from 3 min (99% A, 1% B) to 23 min (30% A, 70% B).

At 20.7 min, the capsid monomer of the CCMV elutes. No significant peaks of impurities seem to be present in the chromatogram. Purity was calculated by the relative peak areas. The chromatographic analysis software Chromeleon (version 7.2.10.23925) was used for peak picking and integration, leading to a relative area of 98.4% for the CCMV peak. For the full report of the analysis, please refer to Figure S5 in the supplementary information.

### 3.8. Determination of Particle Integrity and Size Distribution

**Figure 12:**
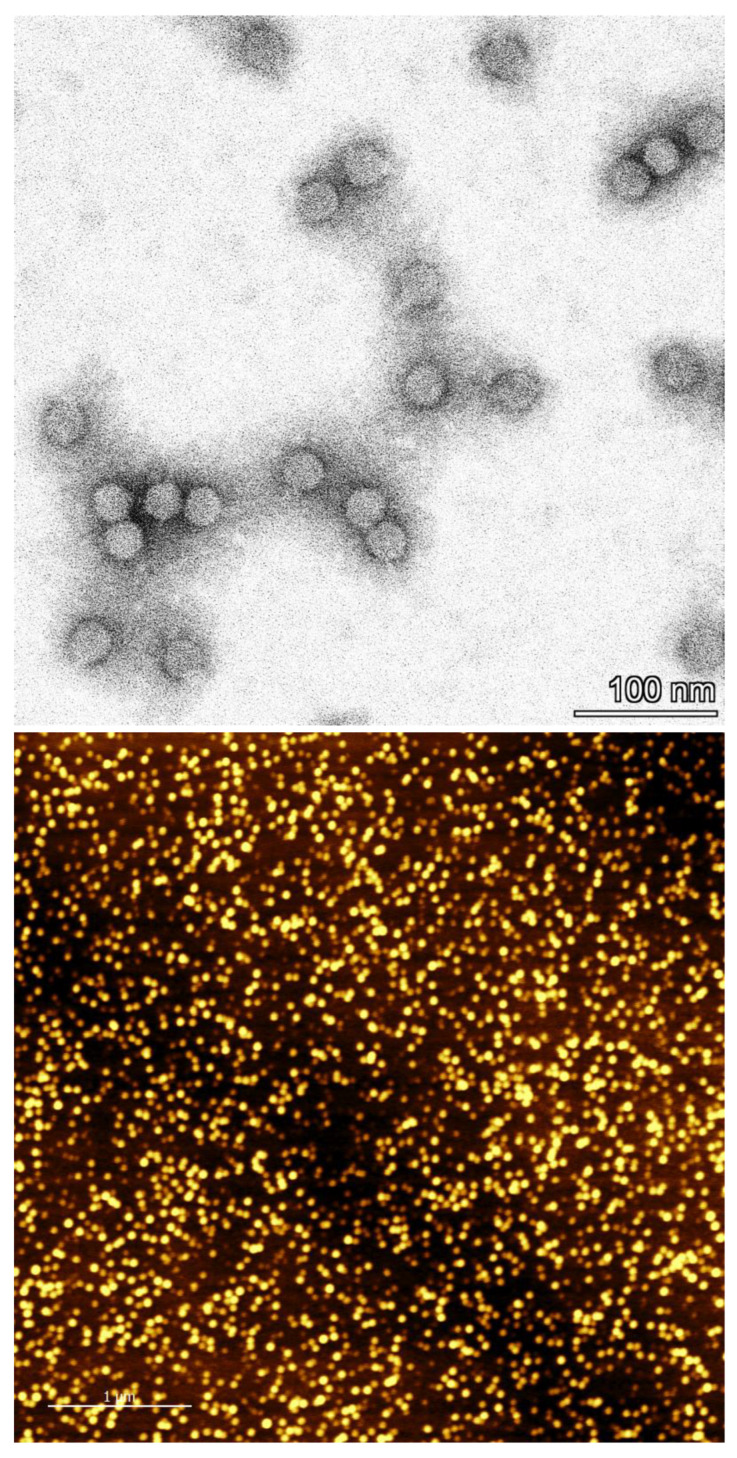
Representation of CCMV nanoparticles after affinity extraction. **Top**: Negative staining TEM image showing virions with a diameter of 28 nm. **Bottom**: AFM image.

For a quick purity assessment and determination of particle integrity, the absorbance ratio of 260/280 nm may be used. This ratio reflects the amount of RNA and protein in the sample and should be between 1.5 and 1.7 according to the literature [60]. In the case of sample 5 (the affinity-purified CCMV), this ratio was 1.6. The absorption spectrum is shown in Figure S6 in the supplementary information. However, care should be taken with the interpretation of such absorption spectra. As shown in Figure 10, certain impurities, such as PEG, can only be detected using more sophisticated methods—for example, mass spectrometry. Still, the absorbance ratio is useful to quickly detect the right RNA/protein ratio in the sample without much effort. In addition, transmission electron microscopy (TEM) and atomic force microscopy (AFM) were used to confirm that the CCMV isolated from the affinity extraction is present in the form of intact and monodisperse virions (Figure 12). Dynamic light scattering (DLS) experiments also confirmed intact and monodisperse particles (Supplementary Materials Figure S7).

### 3.9. Quantification of CCMV by ELISA

The yield of CCMV after each purification step in the presented protocol was determined by a sandwich enzyme-linked immunosorbent assay (ELISA). For this purpose, polyclonal and monoclonal antibodies were developed.

A polyclonal antiserum against the CCMV (BAM-CCMV-rab-pAb01) was raised by immunization of rabbits with native CCMV as the immunogen. After 63 days, IgG was purified from the final bleed by protein G affinity extraction. The antibody titer was determined using dilutions of the unpurified antiserum, resulting in extraordinarily high titers (Supplementary Materials Figure S8). Even a serum dilution of 1:100,000,000 was still distinguishable from the blank (pre-immune serum). Additionally, monoclonal antibodies were developed by immunization of mice with CCMV. Hybridoma culture supernatants were screened against native CCMV in both PBS pH 7.4 and sodium acetate buffer pH 4.8, and a promising clone, BAM-CCMV-29-81, was found and subcloned. As proposed in earlier works [63], a peptide mass fingerprint of BAM-CCMV-29-81 was generated, which is shown in Figure S9 in the supplement. These spectra and the open-source software ABID 2.0 (https://bam.de/ABID, accessed on 28 February 2023) can be used as a future sequence-independent and fast confirmation of identity for this and other antibodies [64].

**Figure 13:**
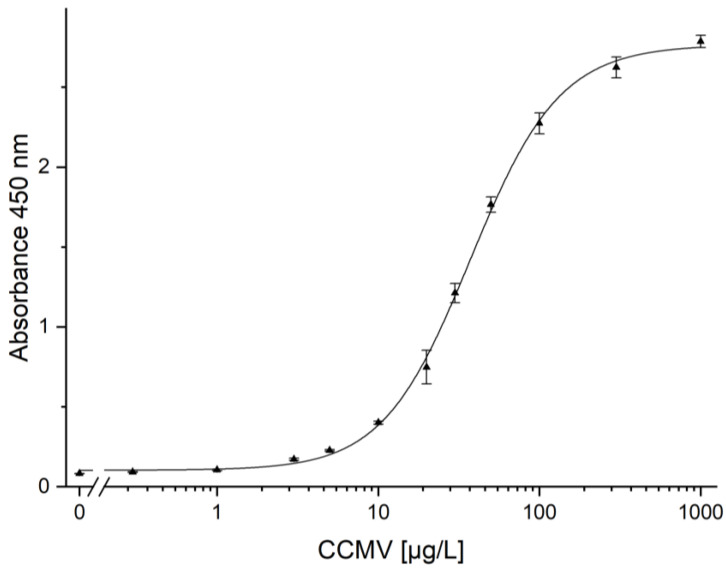
Calibration curve for the determination of CCMV with a sandwich enzyme-linked immunosorbent assay (ELISA). Error bars correspond to the standard deviation of quadruplicates. LOD and LOQ were calculated to be 0.25 µg/L, and 0.79 µg/L, respectively. No matrix effect was observed by diluting the samples to the working range of this assay (3–200 µg/L).

For the immunoassay, a microtitration plate is prepared as follows: Coating with anti-mouse antibody; binding of the monoclonal mouse BAM-CCMV-29-81; blocking with skimmed milk powder; binding of CCMV; binding of polyclonal rabbit anti-CCMV antibody; binding of the peroxidase-conjugated anti-rabbit-IgG antibody, and, finally, colorimetric detection at 450 nm after addition of the TMB substrate and finally H_2_SO_4_ to stop the reaction. The calibration curve measured with the known concentration of pure CCMV is shown in Figure 13. To determine the limits of detection (LOD) and quantification (LOQ), a linear regression of the lowest five test points (0–5 ng/mL) was performed (Supplementary Materials Figure S10). A LOD of 0.25 µg/L and a LOQ of 0.79 µg/L were calculated. Using the calibration curve in Figure 13, a working range from 3 µg/L to 200 µg/L can be defined. Usually, the dilution of the crude plant extract is required. This is beneficial in most cases, as it highly reduces potential interferences. Dilutions of the crude extract of 100-fold or greater were found to show no matrix effects.

By measuring the concentration of CCMV in samples ①, ②, ③, ④, and ⑤, the yield of CCMV after each purification step was determined (see Table 1). The data confirm that the precipitation with PEG 8000 leads to a loss of only ~16% of CCMV from the crude extract. We assume that most of the loss is caused by the co-precipitation of the CCMV with solids after resuspension of the PEG pellet. The affinity purification leads to a loss of about 50% relative to the PEG pelleting step.

## 4. Discussion

The results of this work highlight the power of affinity extraction as a polishing step for the purification of CCMV from crude plant extracts. As SDS-PAGE (Figure 8) and SEC (Figure 9) analyses show, the precipitation of CCMV with PEG is not sufficient to separate the virus from other proteins of the plant matrix. Even worse, PEG remains as an impurity after resuspension of this pellet, as can be seen in the MALDI-TOF MS (Figure 10). Therefore, the final step of every plant virus purification protocol involving precipitation with PEG should also remove this contaminant. So far, many established protocols use density gradient centrifugation for this purpose. However, the ultracentrifuges needed for this procedure are expensive. Furthermore, whether PEG is removed completely at this step remains questionable. Other contaminants may be introduced by sucrose or CsCl gradients. The new purification method by affinity extraction using a novel peptide aptamer can address previous shortcomings of virus purity. No expensive equipment is needed for the workflow, which enables more laboratories to explore plant viruses like CCMV as a potential nanotechnological platform. In the future, we expect that using peptide aptamer-based purification protocols will pave the way for future applications of the CCMV and other plant viruses and ensure that high-purity preparations become readily available. Affinity columns also show promise for the purification of the CCMV coat proteins from culture supernatants from recombinant expression. In that case, a His-Tag would not be needed to purify the coat proteins. The novel peptide aptamer found in this study might also be explored for selective cargo loading and “plug-n-play functionalization” as analogously described for the related plant virus, cowpea mosaic virus (CPMV) [65]. Further modeling of the interaction between the peptide and the CCMV is of interest to determine the exact binding site.

The purity of the CCMV isolates was previously given as an absorbance ratio of A_260/280_, which should be between 1.5 and 1.7. Indeed, this ratio is useful to quickly assess the correct RNA and capsid protein ratio for relatively pure samples. However, today, this approach seems outdated and too imprecise to be considered a valid method for protein purity assessment. Instead, we propose the use of a standard reversed-phase chromatographic separation with detection at 220 nm (see Figure 11) to obtain a purity based on the relative peak ratios of the capsid protein and any contaminants. The correct integrity of the particles should then be checked using methods for particle analysis, such as TEM, AFM, or DLS (see Figure 12).

The quantification of the CCMV in complex matrices can be achieved using immunoassays. This work presents a new ELISA for the CCMV, using both polyclonal and monoclonal antibodies (Figure 13). This assay can be used to determine the concentration of CCMV in field samples. In addition, yields of CCMV for other purification protocols can be evaluated. The monoclonal antibody, BAM-CCMV-29-81, presented here, might also be a good starting point for developing lateral flow tests against the CCMV in plant samples, which can be envisioned to reduce crop failures substantially.

## 5. Conclusions

This work presents a modern protocol for the efficient purification of CCMV from infected leaves involving a novel peptide aptamer for affinity extraction. After harvesting, the leaves are homogenized with a buffer to obtain a crude extract. Subsequently, the virus is precipitated with PEG 8000 by incubation overnight. The virus is then isolated from the PEG-isolate by a CCMV-binding peptide immobilized on a monolithic glass column. The affinity extraction avoids the use of expensive and complex ultracentrifuges, which imposes a severe limitation on the broad application of the CCMV as a nanotechnological platform. Our results show that the final virus isolate is free of any PEG contaminants and has an extraordinary purity of 98.4%, as determined by HPLC at 220 nm. In addition, we presented an immunoassay using a novel monoclonal antibody against the CCMV to monitor the CCMV yield in the different purification steps. The purification protocol presented in this paper considerably reduces the hurdle for scientists outside the phytopathology community to using the CCMV as a nanotechnological platform. Furthermore, the analogous purification of other plant viruses is conceivable using peptide aptamers, which can be obtained without much effort by a new chip-based peptide screening method [43].

## Figures and Tables

**Table 1:** The yield of CCMV after each purification step: absolute values in mg; relative values in per cent relative to 100% determined for the crude extract. Errors were taken from quadruplicate absorbances, calculating the concentrations from the calibration function and determining their standard deviation.

Purification Step	Absolute Yield [mg]	Relative Yield [%]
①—Crude extract	3.52 ± 0.27	100 (def.)
②—Supernatant of 1	0.040 ± 0.001	(1.1)
③—Resuspended pellet	2.91 ± 0.04	83
④—Flowthrough of affinity column	<0.001	(0)
⑤—Eluate of affinity step	1.57 ± 0.02	**45** ^1^

^1^ Final yield, relative to the amount of CCMV in the crude extract.

## Data Availability

Not applicable.

## Supplementary Materials

The following supporting information can be downloaded at: https://www.mdpi.com/article/10.3390/v15030697/s1. Figure S1: Convergence test of the implied timescales for MSM of the peptide aptamer; Figure S2: Free energy surface of the first two independent components from TICA analysis of the peptide aptamer; Figure S3: Western Blot of partially degraded capsid protein; Figure S4: MALDI-TOF mass spectra of partially degraded CCMV isolated from aging leaves; Figure S5: Chromatographic report for purity determination of CCMV; Figure S6: Absorption spectrum of CCMV; Figure S7: Dynamic light scattering of CCMV; Figure S8: Titer determination of rabbit antiserum against CCM; Figure S9: Peptide Mass Fingerprint of BAM-CCMV-28-81 for identity confirmation; Figure S10: Calculation of LOD and LOQ for CCMV immunoassay. Reference [64] is cited in Supplementary Materials.
