# Supplementary material for: Efficient Purification of Cowpea Chlorotic Mottle Virus by a Novel Peptide Aptamer
Source: Viruses. 2023 Mar 7;15(3):697. doi: 10.3390/v15030697 (PMC10051510; doi:10.3390/v15030697)
Supplement: Supplementary file 1 [file viruses-15-00697-s001.zip › viruses-2211385-suppl-conv.pdf]

viruses

MDPI

Supplementary materials

Efficient Purification of Cowpea Chlorotic Mottle Virus by a Novel Peptide Aptamer

Georg Tscheuschner [1], Marco Ponader [1], Christopher Raab [1], Prisca S. Weider [1], Reni Hartfiel [1], Jan Ole Kaufmann [1,2,3], Jule L. Völzke [1], Gaby Bosc-Bierne [1], Carsten Prinz [1], Timm Schwaar [4], Paul Andrle [1], Henriette Bäßler [1], Khoa Nguyen [1], Yanchen Zhu [5], Antonia S. J. S. Mey [5], Amr Mostafa [6], Ilko Bald [6] and Michael G. Weller [1,*]

[1] Federal Institute for Materials Research and Testing (BAM), 12489 Berlin, Germany
[2] Charité—Universitätsmedizin Berlin, Corporate Member of Freie Universität Berlin, Humboldt-Universität zu Berlin, and Berlin Institute of Health, 10117 Berlin, Germany
[3] Department of Diagnostic and Interventional Radiology, Technical University of Munich, 81675 Munich, Germany
[4] SAFIA Technologies GmbH, 12489 Berlin, Germany
[5] EaStCHEM School of Chemistry, University of Edinburgh, Edinburgh EH9 3FJ, UK
[6] Institute of Chemistry—Physical Chemistry, University of Potsdam, 14476 Potsdam, Germany

Citation: Tscheuschner, G.; Ponader, M.; Raab, C.; Weider, P.S.; Hartfiel, R.; Kaufmann, J.O.; Völzke, J.L.; Bosc-Bierne, G.; Prinz, C.; Schwaar, T.; et al. Efficient Purification of Cowpea Chlorotic Mottle Virus by a Novel Peptide Aptamer. *Viruses* **2023**, *15*, 697. https://doi.org/10.3390/v15030697

Academic Editor: Fei Yan

Received: 26 January 2023
Revised: 28 February 2023
Accepted: 1 March 2023
Published: 7 March 2023

Copyright: © 2023 by the authors. Licensee MDPI, Basel, Switzerland. This article is an open access article distributed under the terms and conditions of the Creative Commons Attribution (CC BY) license (https://creativecommons.org/licenses/by/4.0/).

1. Peptide modeling with additional information from the Markov State Model

Figure S1 illustrates the implied timescales from the 5 μs trajectory of MD simulations. The plot was used to determine the 1ns lag time for the building of the MSM and the coarse-graining into two dominant states, as there is one slow time scale observed in the system (blue line). Figure S2 shows the projection onto two dominant independent components from the TICA analysis.

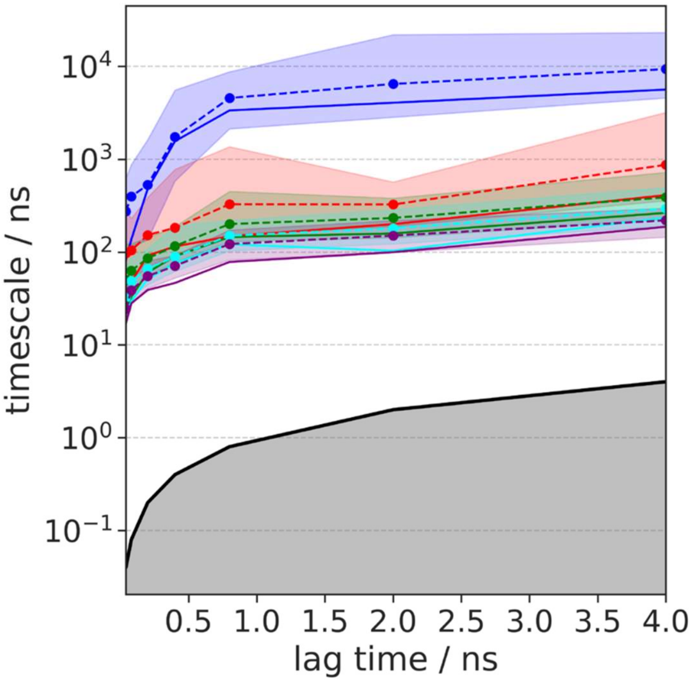

Figure S1. The convergence test of the implied timescales associated with the five slowest processes. Solid lines refer to the timescales estimated from the maximum likelihood MSM. Dashed lines refer to the ensemble means computed from Bayesian sampling, with shaded areas indicating the 95 % confidence intervals. The black line refers to the equality of timescale and lag time. The lag time was chosen to be 1 ns for estimating MSM, as most implied timescales converge at this lag time. The relatively large gap between the slowest (blue) and the second slowest (red) timescales suggests two metastable states.

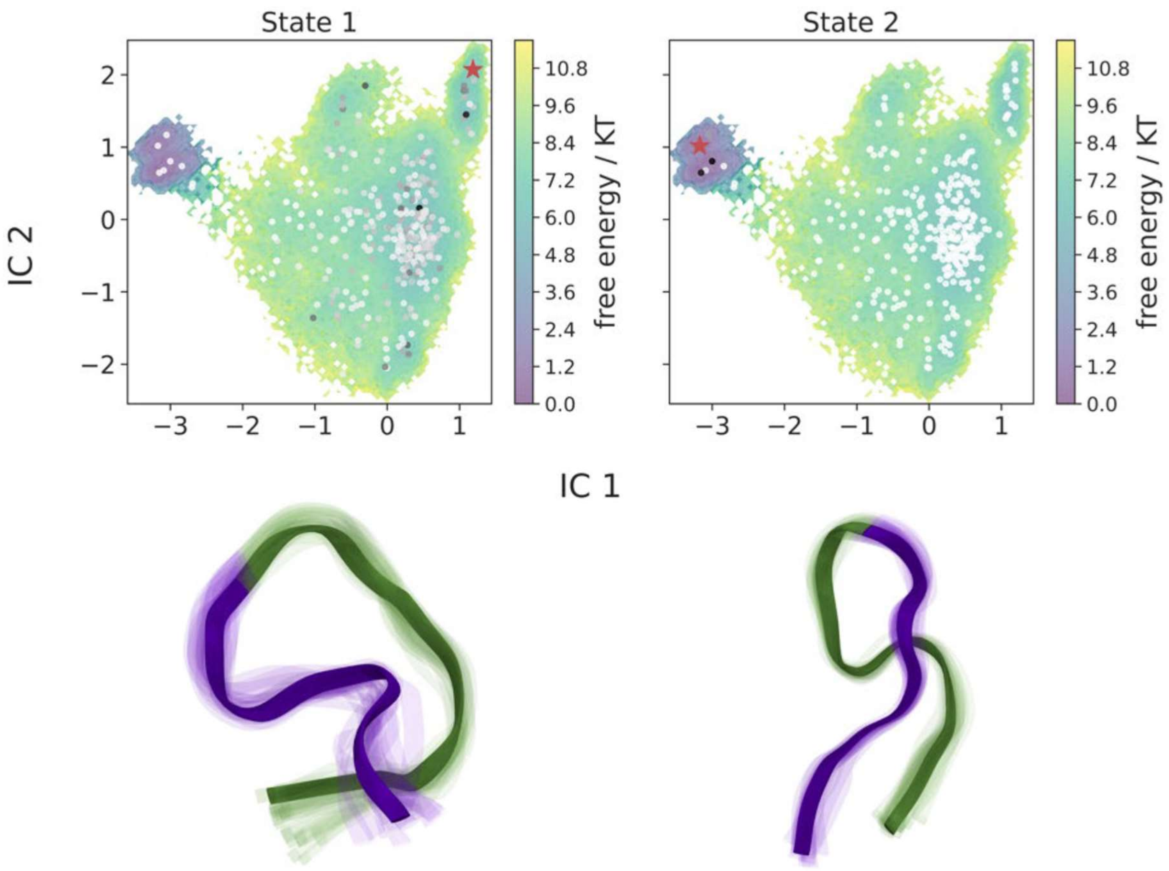

Figure S2. Top: the free energy surface shown on the first two independent components (IC1 and IC2) with microstates colored according to the metastable distribution of metastable state 1 (left) and state 2 (right). The dark color indicates a high probability of metastable assignment. Red stars refer to the centers of metastable states. The populations of metastable states 1 and 2 are 3.4 % and 96.6 %, respectively, computed as the sum of their microstate populations. Bottom: the ensembles of 20 conformations sampled from metastable states 1 (left) and 2 (right). The linker is shown in purple, and the aptamer is shown in green. More details of MSM estimation and analysis can be found at: https://github.com/meyresearch/CCMV_aptamer_msm (accessed on 28 February 2023).

2. Degradation of CCMV due to aging leaves

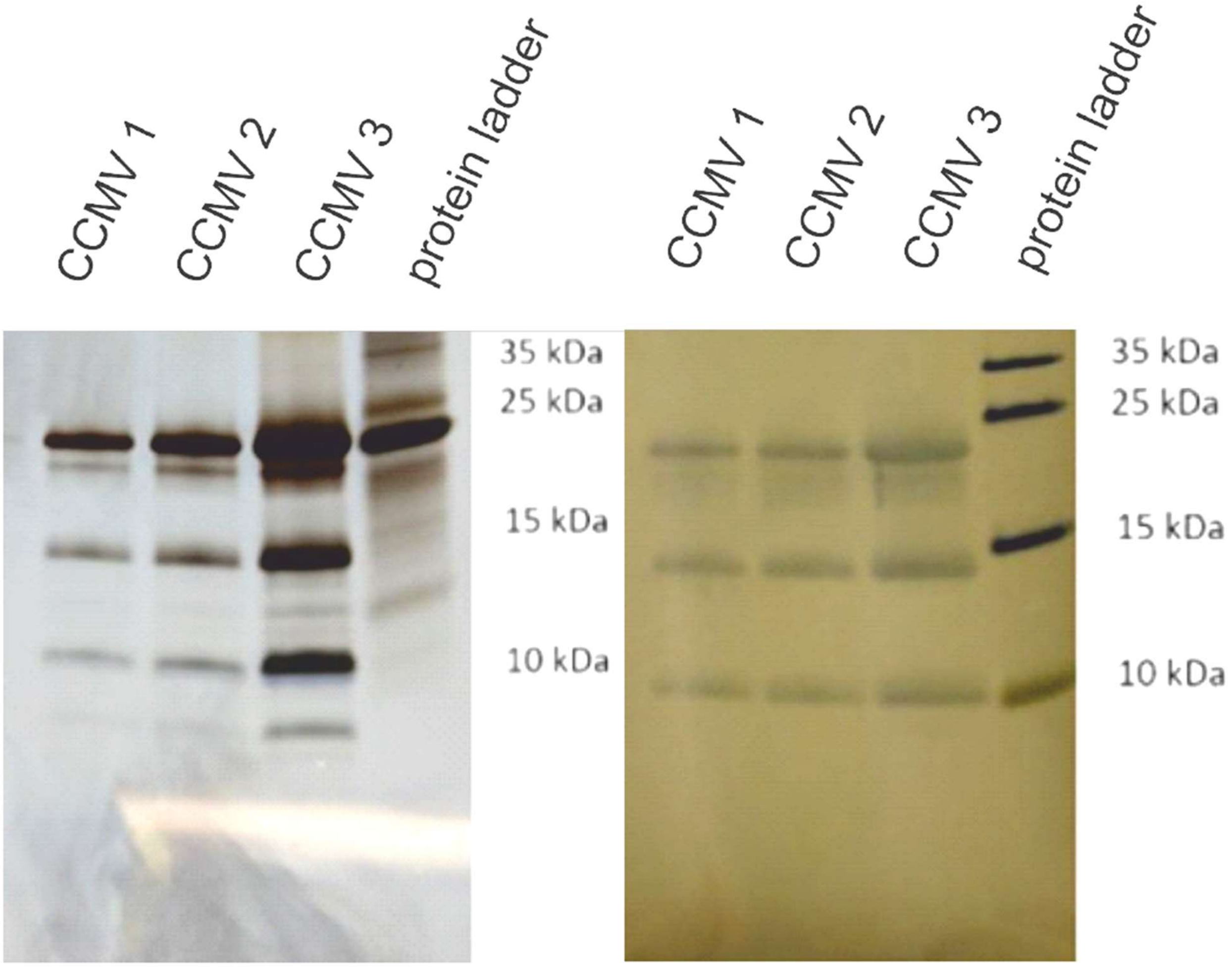

Figure S3. Silver-stained SDS-PAGE (left) of partially degraded CCMV and Western-Blot (right) with peroxidase-conjugated mAb BAM-CCMV-29-81. The Western Blot shows that the bands seen in the SDS-PAGE gel all belong to the CCMV capsid protein. The older the leaves from the purified CCMV (1-3 in ascending age: 3, 6, and 9 weeks), the more fragmentation of the capsid protein takes place. The CCMV coat protein has a mass of ~20 kDa. Its degradation products are visible in the bands corresponding to masses lower than that.

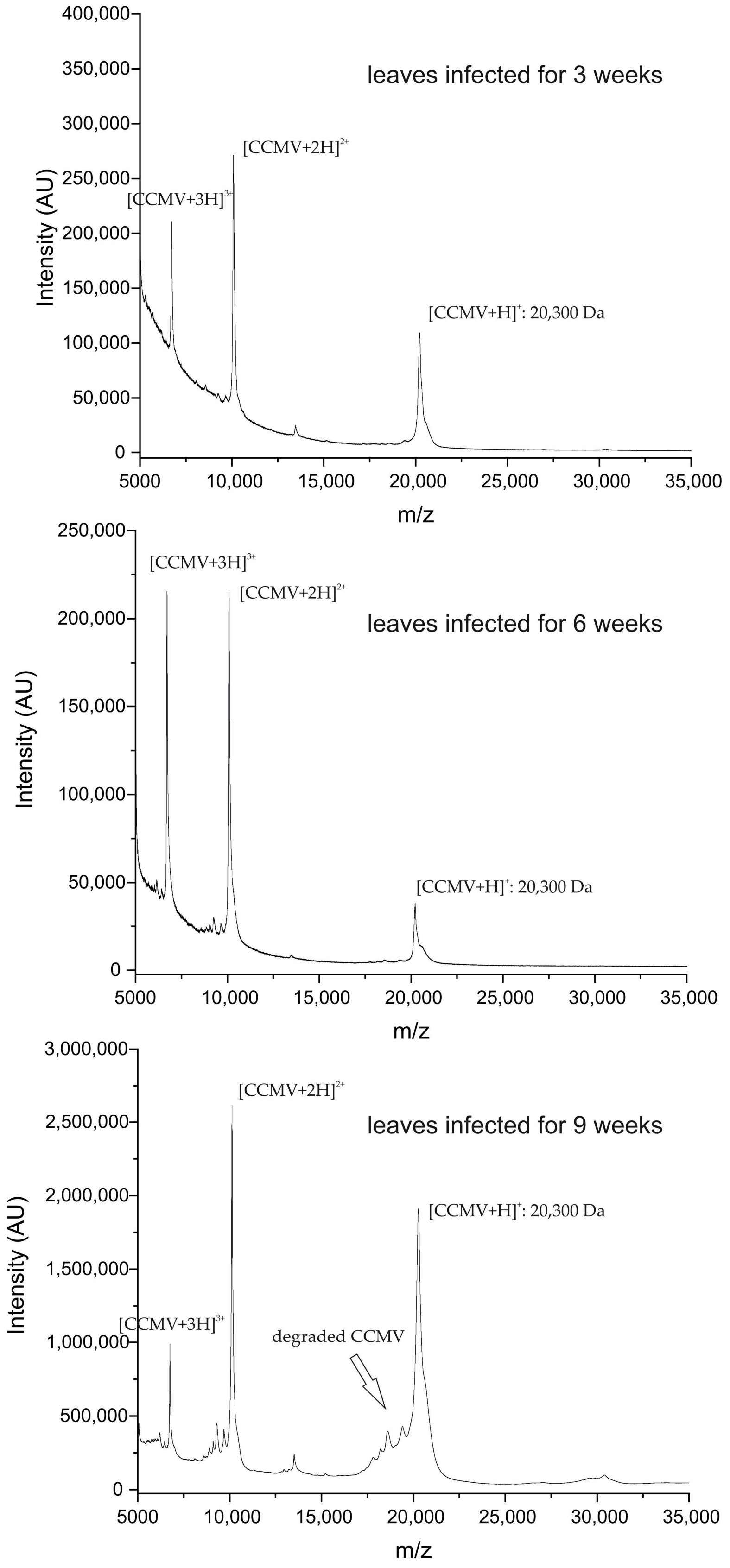

Figure S4. MALDI-TOF mass spectra of CCMV with varying degrees of proteolytic degradation depending on the time that the virus was allowed to propagate in the leaves until harvested. After 9 weeks of being infected, leaves yielded significantly higher amounts of degraded capsid proteins, as can be seen from multiple fragments smaller than 20,300 Da and their respective multiply charged species.

3. Determination of CCMV purity by reversed-phase chromatography (HPLC) and detection at 220 nm

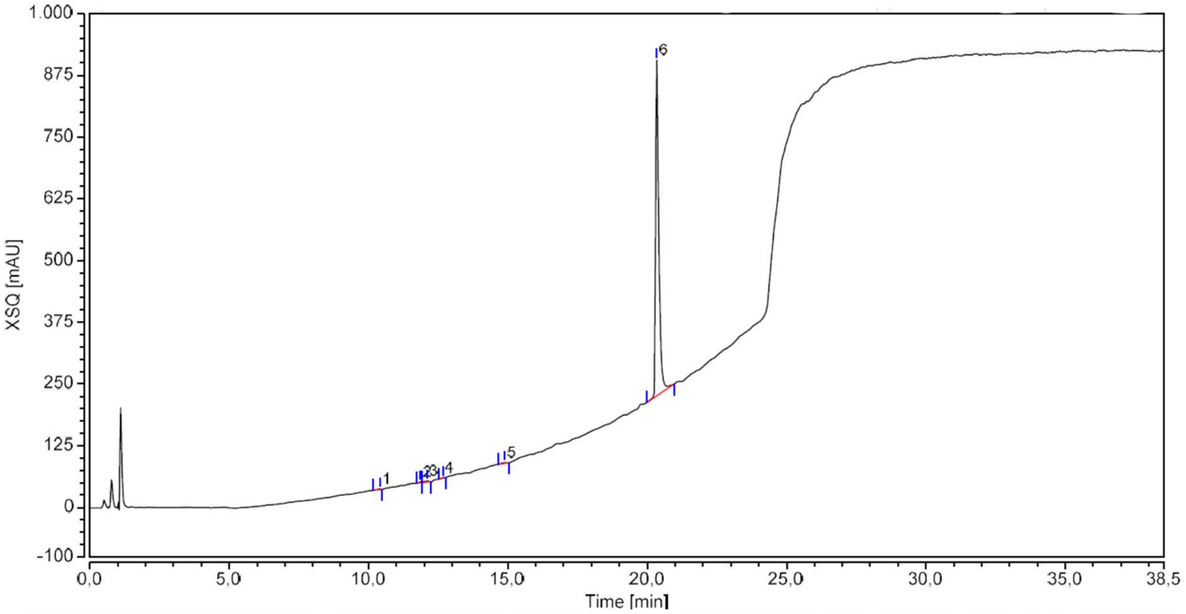

No.	Retention Time min	Area mAU*min	Height mAU	Relative Area %	Relative Height %	Peak Width min
1	10,407	0,210	1,156	0,23	0,17	0,24
2	11,854	0,208	1,955	0,23	0,28	0,21
3	12,101	0,570	2,608	0,62	0,38	0,56
4	12,667	0,151	1,104	0,16	0,16	0,30
5	14,881	0,334	1,530	0,36	0,22	0,34
6	20,341	90,282	679,853	98,39	98,79	0,19
**Total:**		**91,756**	**688,207**	**100,00**	**100,00**	

Figure S5. Summary of the report from the chromatographic analysis software Chromeleon (version 7.2.10.23925) for the determination of CCMV purity with detection at 220 nm. For peak picking and integration during gradient separation from 3 to 23 minutes, the default settings of the software were used. The software finds 6 peaks in total and gives a relative peak area of 98.39% for CCMV in this sample.

4. Determination of particle integrity

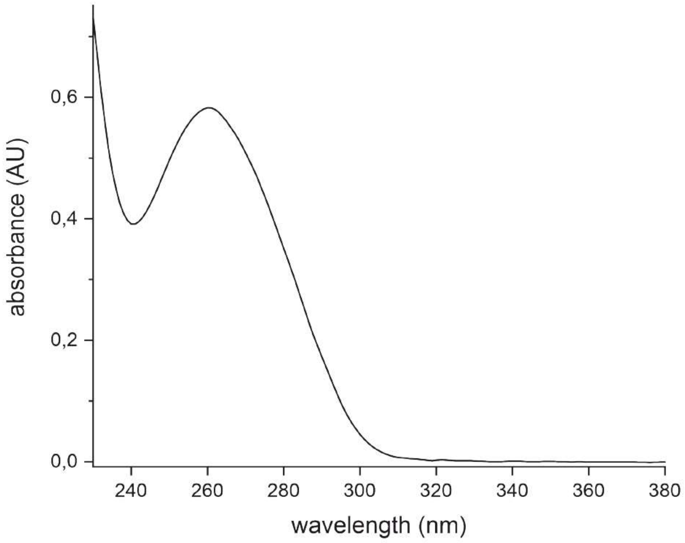

Figure S6. Absorption spectrum of CCMV after affinity purification with peptide aptamer. The absorbance ratio of 260/280 nm gives 1.6.

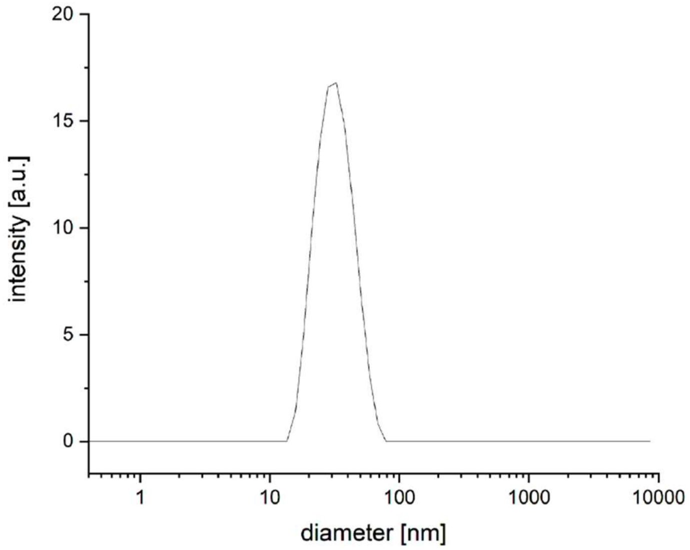

Figure S7. Dynamic light scattering measurements show a narrow distribution of particles with a mean size of 32.7 nm, which is in good agreement with TEM and AFM data. Note that DLS measurements include the hydration sphere of the particles.

5. Titer of anti-CCMV rabbit serum

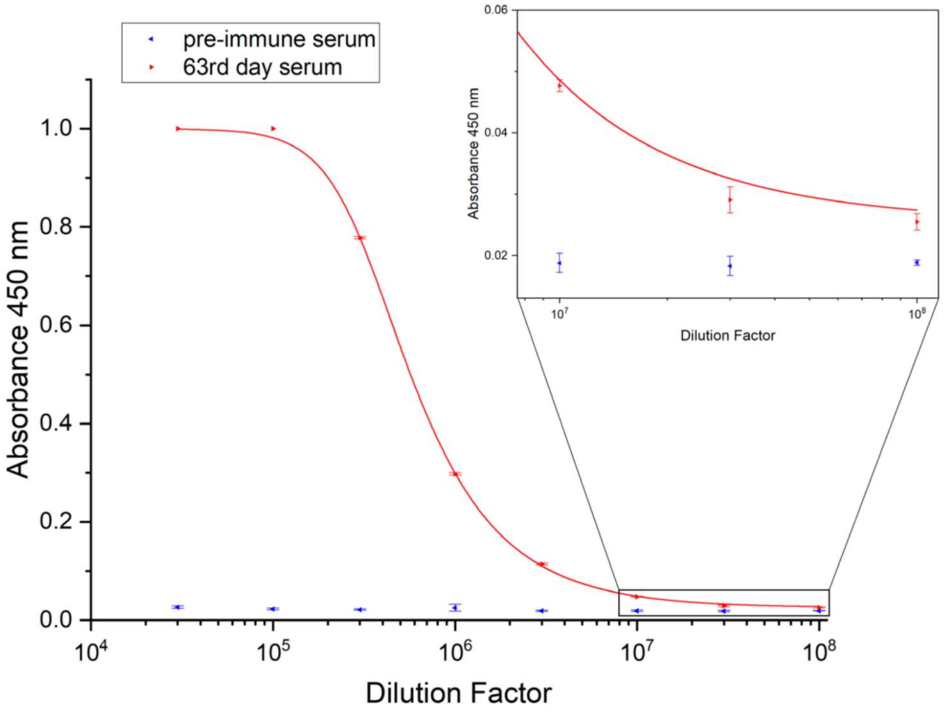

Figure S8. Titer determination of rabbit antiserum against CCMV. Microplates were coated with CCMV. Subsequently, CCMV antiserum (red) and pre-immune serum (blue) were added at different dilutions. Peroxidase-conjugated anti-rabbit antibodies were used for detection. The resulting titer is at least 1:100,000,000, as signals are still distinguishable from blank measurements with pre-immune serum at this dilution.

6. Peptide Mass Fingerprints of BAM-CCMV-29-81 for identity confirmation

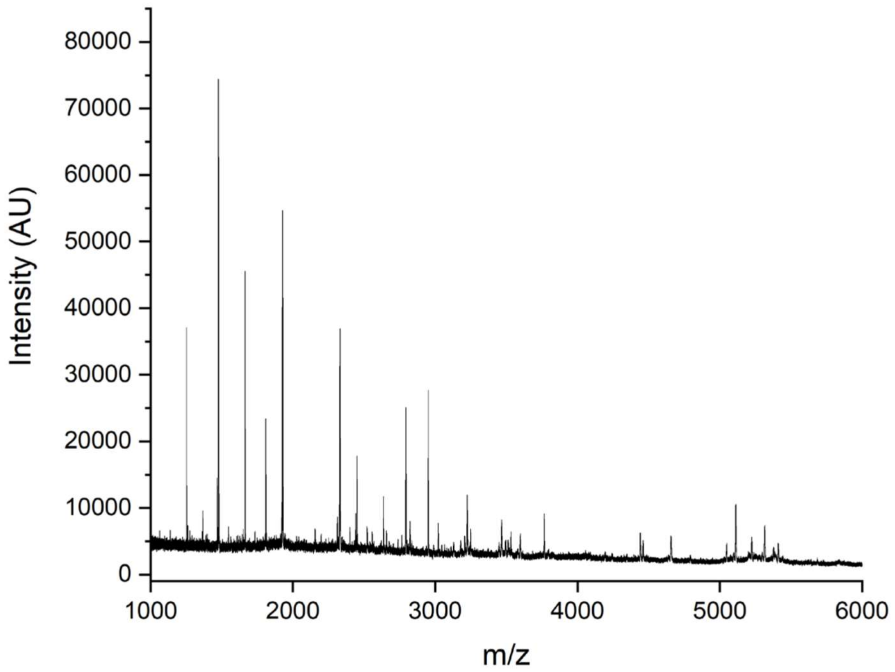

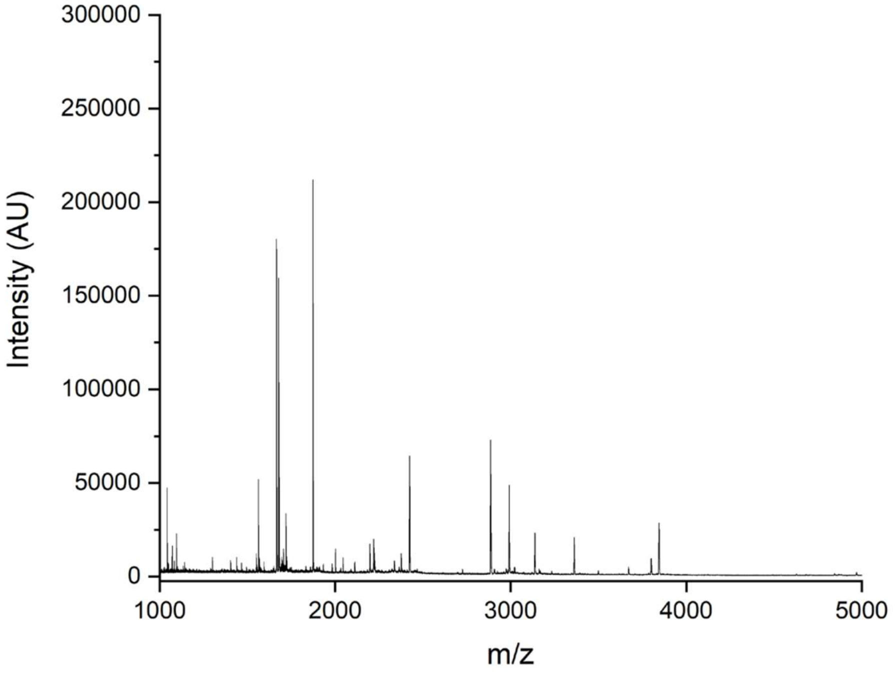

Figure S9. Peptide Mass Fingerprint of BAM-CCMV-29-81 with MALDI-TOF-MS after partial acidic hydrolysis (top) and tryptic digestion (bottom). Antibody cleavage was performed according to Tscheuschner et al., 2022 [64]. The raw data of the fingerprint spectra (see electronic supplement) may be used for future comparison for identity confirmation using the open-source software ABID 2.0 (https://bam.de/ABID, last accessed 28 February 2023).

7. Estimation of LOD and LOQ of CCMV immunoassay

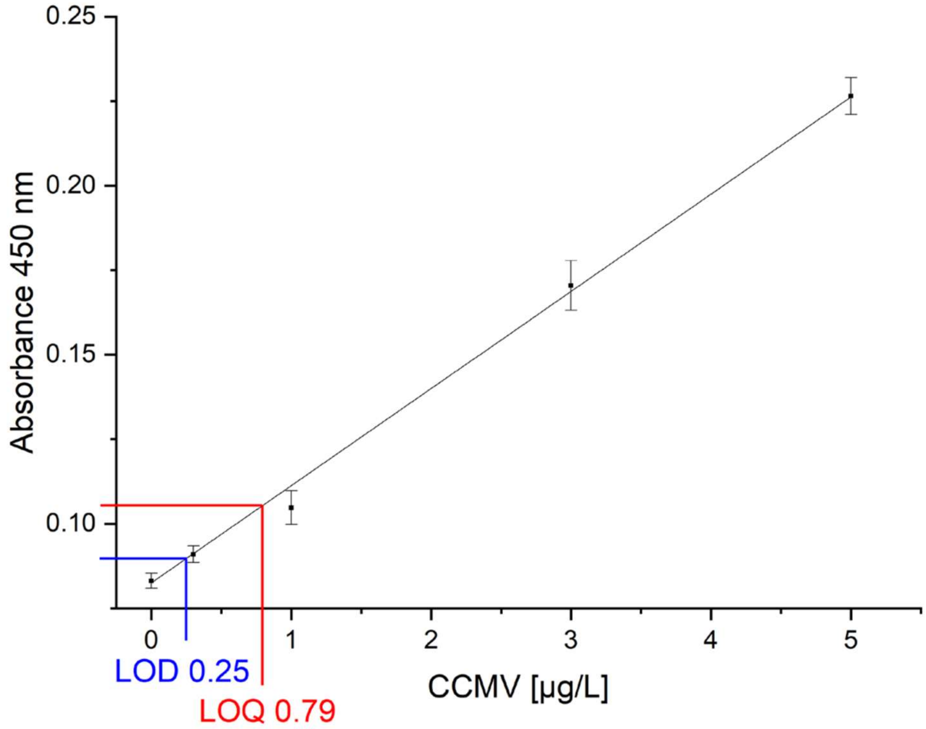

Figure S10. Linear regression of the lowest five test points of the immunoassay calibration curve. LOD and LOQ were determined as y_b+3S_b and y_b+10S_b, respectively, where y_b is the average absorption of 26 blanks and S_b is its standard deviation. LOQ and LOQ absorption values (y) were translated to concentration values (x) with the linear calibration function above using the following fit: x = (y – 0.0823 ± 0.00065)/(0.029 ± 0.00037). For the LOD, 0.25 µg/L, and for the LOQ, 0.79 µg/L were determined.
